# Prognostic Factors for Physical Functioning After Multidisciplinary Rehabilitation in Patients With Chronic Musculoskeletal Pain: A Systematic Review and Meta-Analysis

**DOI:** 10.1097/AJP.0000000000000669

**Published:** 2018-10-26

**Authors:** Elena Tseli, Katja Boersma, Britt-Marie Stålnacke, Paul Enthoven, Björn Gerdle, Björn O. Äng, Wilhelmus J.A. Grooten

**Affiliations:** * Department of Neurobiology, Care Sciences and Society, Division of Physiotherapy, Karolinska Institutet; § Department of Clinical Sciences, Danderyd Hospital, Karolinska Institutet; Department of Rehabilitation Medicine, Danderyd Hospital; †† Functional Area Occupational Therapy & Physiotherapy, Allied Health Professionals Function, Karolinska University Hospital, Stockholm, Sweden; ‡ Department of Community Medicine and Rehabilitation, Rehabilitation Medicine, Umeå University, Umeå; † School of Law, Psychology and Social Work, Örebro University, Örebro; ∥ Department of Medical and Health Sciences; ¶ Department of Medical and Health Sciences, Pain and Rehabilitation Centre, Linköping University, Linköping; # School of Education, Health and Social Studies, Dalarna University; ** Center for Clinical Research Dalarna,Uppsala University, Falun, Sweden

**Keywords:** chronic musculoskeletal pain, GRADE, interdisciplinary rehabilitation, meta-analysis, prognostic factors, treatment outcome

## Abstract

**Objectives:**

This systematic review aimed to identify and evaluate prognostic factors for long-term (≥6 mo) physical functioning in patients with chronic musculoskeletal pain following multidisciplinary rehabilitation (MDR).

**Materials and Methods:**

Electronic searches conducted in MEDLINE, PsycINFO, EMBASE, CINAHL, Web of Science, and Cochrane CENTRAL revealed 25 original research reports, published 1983-2016, (n=9436). Potential prognostic factors relating to initial pain and physical and psychological functioning were synthesized qualitatively and quantitatively in random effects meta-analyses. The level of evidence (LoE) was evaluated with Grading of Recommendations Assessment, Development and Evaluation (GRADE).

**Results:**

*Pain-related factors* (intensity and chronicity) were not associated with function/disability at long-term follow-up, odds ratio (OR)=0.84; 95% confidence interval (CI), 0.65-1.07 and OR=0.97; 95% CI, 0.93-1.00, respectively (moderate LoE). A better function at follow-up was predicted by *Physical factors*; higher levels of initial self-reported functioning, OR=1.07; 95% CI, 1.02-1.13 (low LoE), and *Psychological factors*; low initial levels of emotional distress, OR=0.77; 95% CI, 0.65-0.92, low levels of cognitive and behavioral risk factors, OR=0.85; 95% CI, 0.77-0.93 and high levels of protective cognitive and behavioral factors, OR=1.49; 95% CI, 1.17-1.90 (moderate LoE).

**Discussion:**

While pain intensity and long-term chronicity did not predict physical functioning in chronic pain patients after MDR, poor pretreatment physical and psychological functioning influenced the prognosis negatively. Thus, treatment should further target and optimize these modifiable factors and an increased focus on positive, psychological protective factors may perhaps provide an opening for yet untapped clinical gains.

## Abstract 2

Supplemental Digital Content is available in the text.

Chronic musculoskeletal pain (ie, pain duration >3 mo) such as chronic neck/shoulder and back pain, or generalized widespread pain, is a major health and socioeconomic burden. Although etiology, localization, and diagnoses might differ, chronic pain itself could be considered a disease in its own right.^1^ About a quarter of the adult population live with chronic pain of significant intensity,^2^,^3^ which may result in poor health including psychological distress, reduced quality of life, impaired physical functioning, reduced work ability, and increased sick leave.^4^

From a therapeutic perspective, chronic musculoskeletal pain is a complex, multifaceted condition. A biopsychosocial approach is necessary for understanding and treating chronic pain—as a result, a comprehensive, multimodal and interdisciplinary, pain management method, here referred to as multidisciplinary rehabilitation (MDR, also known as interdisciplinary rehabilitation,^5^ multimodal rehabilitation, and multimodal pain therapy) is advised for this patient group. On the basis of a cognitive-behavioral therapy approach, it incorporates education, physical activity and exercise, coping skills, and occupational therapy sessions in a multimodal rehabilitation program. MDR is administered by multidisciplinary teams, which commonly include physicians, psychologists, physiotherapists, occupational therapists, social workers, and other health professionals. The team’s collaboration in assessment and shared goal-setting is an essential component, adding value beyond the effects of the multiple modalities provided in pain treatment.^6^ Existing data shows that MDR is effective compared with single-treatment or treatment-as-usual programs, but the effects are at best moderate and need further study.^7^–^13^

Studying effectiveness and effect moderators of MDR in patients with chronic musculoskeletal pain has been recognized as a major challenge. The complexity of the various pain conditions and the complexity of the intervention itself,^14^ accompanied by the lack of a standardized, internationally accepted definition of the treatment, hinder comparative clinical trials and meta-analyses,^15^ which delays evidence on how outcomes for this patient group can be optimized. It is, however, believed that outcomes would improve if treatments could be better customized to a patient’s profile, that is the characteristics of their initial biopsychosocial status.^10^,^11^

Prognostic factor research aims to identify factors associated with clinical outcomes to provide data on the likely health outcomes among people with a given health condition. Riley et al^16^ state that prognostic factors can help “inform clinical and therapeutic decisions (either directly or as part of prognostic models for individualised risk prediction)… and help identify targets for new interventions that aim to modify the course of a disease or health condition.” Predictive factor (or predictor) is a term related to the term prognostic factor, and these are sometimes used synonymously. Predictive factor, however, is used more in the context of measures of response to a given therapy among others.^17^ In the present study, we have chosen to use the term prognostic factor or indicator consistently.^18^

Although many clinical studies have performed these analyses to identify factors of importance for future outcomes in patients with chronic musculoskeletal pain, the body of evidence of prognostic factors is still insufficient to predict MDR outcomes.

Rather than looking at any study in isolation, systematic reviews can provide an overview of a whole body of research on a topic—and meta-analyses have the potential to test more rigorously whether there are any systematic indicators with prognostic value. With knowledge of the likely future outcomes, one may identify those who benefit from MDR and those at risk of poor outcome. This could provide ideas on what grounds to tailor clinical practice, and generate ideas for future research in the development of treatment and screening strategies.

Previous systematic reviews of prognostic factor studies on patients following MDR were performed on specific populations based on medical diagnosis, such as fibromyalgia^19^ and low-back pain^20^,^21^; however, heterogeneity of studies and lack of power hindered meta-analyses. Using a qualitative data-synthesis, some prognostic factors were identified: a poorer outcome was predicted by psychological factors, in particular high initial depression^19^ and higher pain intensity and pain interference,^20^ while a positive outcome was predicted by work-related functioning, and active coping skills at baseline.^20^ Some prognostic factors pointed in opposite directions depending on outcome, while sociodemographic factors did not seem to have prognostic value for outcomes, or were inconclusive. The evidence from systematic reviews therefore still remains limited and shows mixed findings. Lately, another approach has been taken in attempts to identify generic prognostic factors across a range of musculoskeletal pain conditions and across a wider timeline, from acute to chronic pain, as well.^22^–^24^ These reviews are well-powered, but none of them have yet targeted patients with chronic pain following MDR programs. A thorough overview of factors that might predict important outcomes following MDR intervention is therefore called for.^9^,^11^ For these reasons, our research team initiated a systematic review with the intent of gathering existing data of possibly important prognostic factors available at pretreatment level, across a number of outcomes that are targeted by the MDR.^25^ In this first part of the systematic reviews, we have focused on physical functioning as the main outcome.

Improving physical functioning and decreasing pain’s interference with functioning are of great clinical importance,^26^,^27^ and these are therefore important targets of MDR. Knowledge of early prognostic indicators of outcome is therefore of great clinical importance as well.

## OBJECTIVE

The aim of this systematic review was to identify, evaluate, and meta-synthesize published data on prognostic factors, related to baseline information on pain and physical and emotional functioning, for physical functioning at least 6 months post MDR in patients with chronic musculoskeletal pain.

## MATERIALS AND METHODS

This systematic review used a random effects meta-analysis of published original research reports with a longitudinal follow-up of early prognostic factors preceding MDR. The review was conducted by an interdisciplinary research team. It conforms to the Preferred Reporting Items for Systematic Reviews and Meta-Analyses (PRISMA) statement,^28^ with particulars of the Meta-analysis Of Observational Studies in Epidemiology (MOOSE) guidelines^29^ in consideration. A protocol with the defined objectives, eligibility criteria, and planned methods of the complete review project was registered in the International Prospective Register of Systematic Reviews (PROSPERO, id:CRD42016025339) at an early stage of the study, and a study protocol reporting the review process was published ahead of the study.^25^

### Data Sources and Search Procedure

Articles published in English between 1980 and April 2017 were identified through systematic electronic searches of 6 reference databases: MEDLINE and PsycINFO (via Ovid), EMBASE (via Elsevier), CINAHL (via EBSCO), Web of Science (via Thomson Reuters), and the Cochrane Central Register of Controlled Trials (CENTRAL). With the support of a research librarian, we developed a comprehensive search strategy combining 4 search parameters; “Chronic Pain”—“Multidisciplinary Rehabilitation”—“Treatment Outcome”—“Prediction,” for inclusion of all common diagnoses of chronic musculoskeletal pain conditions targeted in MDR comprising a follow-up of clinically important outcomes and explorative approaches to all prognostic factors possibly studied. An a priori decision was made to only search for published work. Consistent with the explorative objective, the search was unrestricted except for 2 limitations; publication language and publication date. To identify additional studies, a manual search of reference lists of obtained and relevant articles was conducted. The complete search strategy is described in detail in Supplemental Appendix 1 (Supplemental Digital Content 1, http://links.lww.com/CJP/A533).

### Study Selection

Inclusion criteria were: (1) studies with a longitudinal design, either observational (cohort, case-control) or experimental/clinical trials (randomized controlled trial), (2) studies that investigated prognostic factors of treatment outcome, (3) in adults aged 18 to 67 years (ie, the working-age population), with a chronic musculoskeletal pain condition; defining chronic as a duration of >3 months and delimitating musculoskeletal pain conditions to common nonspecific musculoskeletal pain diagnoses such as back pain, neck pain, and generalized pain syndromes (including fibromyalgia and general widespread pain) but not those emanating from, for example, malignancies, systemic or inflammatory diseases (eg, rheumatoid arthritis), or degenerative joint diseases (eg, osteoarthritis-related joint pain), (4) studies on patients that had taken part in multidisciplinary/interdisciplinary/multimodal rehabilitation following the biopsychosocial model^6^ and coordinated by ≥3 different health professionals. MDR could be of any duration/intensity and rehabilitation approach, in inpatient or outpatient settings, (5) studies on interventions that targeted core outcome domains as recommended by the Initiative on Methods, Measurement, and Pain Assessment in Clinical Trials (IMMPACT), and reported results on either pain, physical functioning, work ability, or health-related quality of life (QoL) and emotional functioning.^30^ Outcome measures were allowed to vary as long as they could be grouped under the domains of interest, (6) studies with a follow-up of 6 months or longer were set as a minimum time criterion for analysis of clinically relevant long-term outcomes, and (7) only original research reports in peer-reviewed journals, published in English, and in full text were eligible.

Studies were excluded if: (1) they lacked a presentation of data from baseline to a follow-up of at least 6 months in the prediction analyses, or (2) they investigated the process of change as a prognostic factor, that is the actual changes occurring during treatment as prognostic indicators of outcome. Eligibility criteria were defined as PICOT (ie, Population Intervention/Variable of Interest Comparator Outcome and Time).^25^

**FIGURE 1:**
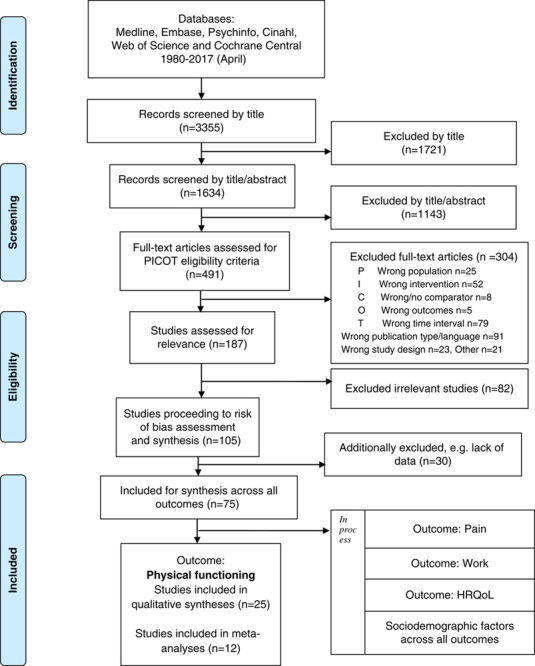
PRISMA flow chart of study selection. HRQoL indicates health-related quality of life; PRISMA, Preferred Reporting Items for Systematic Review and Meta-Analysis.

The study selection procedure was performed in the Covidence online systematic review platform^31^ and a PRISMA flow diagram^32^ was used to document the flow of included and excluded studies, along with the reasons for exclusion (Fig. 1). The selection process was performed in 4 steps: (1) screening of titles, (2) screening of abstracts, (3) screening of full texts for PICO eligibility, and (4) screening of full texts for relevance according to study objective. A first raw screening of titles was performed by one reviewer. During the following selection steps, every article was appraised by 2 reviewers independently. The articles were randomly assigned to the reviewer teams. Every step was first piloted to validate the interpretation of the criteria. Interrater agreement throughout the review process was evaluated and agreement ranged from 72% to 83% (Cohen κ=0.342 to 0.648). Disagreements were resolved through discussions with the full review team.

In the current study, further selection was made for papers evaluating the outcome “Physical functioning.” Typically, measures commonly used in clinics assess either the ability for various sorts of functioning, or conversely the inability for functioning, that is disability—thus reflecting opposite perspectives of the same construct (physical functioning). Moreover, only prognostic factors related to initial pain and physical and emotional functioning were included for analysis in the present paper. Sociodemographic factors will be presented elsewhere (Fig. 1).

### Quality Assessment

Articles deemed relevant from the full text screening were assessed for internal validity with The Quality in Prognostic Studies (QUIPS)-tool.^33^ Potential threats to validity were assessed within the 6 domains: (1) study participation, (2) study attrition, (3) prognostic factor measurement, (4) outcome measurement, (5) study confounding, and (6) statistical analysis and reporting, similar to Cochrane’s risk of bias (RoB) assessment, but with emphasis on evaluating critical methodological criteria for bias in prognostic studies as recommended by the Cochrane Prognosis Methods Group. All articles were assessed independently by 2 reviewers: 1 senior reviewer assessed all studies, which were then divided between 2 other researchers in accordance with the randomization scheme. The process was piloted a priori for interrater agreement. The percent agreement ranged between 48% and 81% and the Prevalence and Bias Adjusted Kappa-Ordinal Scale (PABAK-OS) across RoB domains varied between 0.227 and 0.719. Consensus on final ratings per domain was reached through discussions within the team. The QUIPS-file, with the key list for our study’s topic, is available from the author on request.

The overall study quality, pertaining to the outcome for each prognostic factor, was rated as low/moderate/high RoB. The synthesis of the *between-studies* risk of bias (ROB), for overall study quality, was based on thoughtful scrutiny for every outcome as we avoided making a simple summary score. Every outcome was assessed in 2 ways: (1) by classifying each study into 3 levels of RoB based on the ratings of all 6 domains together. We classified a study to have a low RoB when at least 5 of the domains had low RoB and none of the domains had high RoB, to a Moderate RoB when the study had a maximum of 2 moderate RoB and the rest low RoB, and a high RoB study when one or more domains had high RoB or there were 3 domains or more with moderate RoB, (2) RoB was also analyzed across every RoB-domain separately to identify specific problematic areas pertaining to a specific outcome. The analyses of overall study quality were later also incorporated in the Grading of Recommendations Assessment, Development and Evaluation (GRADE) summary, under the factor “study limitations.”

### Data Extraction and Data Syntheses

From each included study, data were collected on: (1) participant and sample characteristics, (2) intervention characteristics, (3) independent variables (potential prognostic factors) and assessment methods, (4) dependent variables (outcome domains) relating to physical functioning (primary outcome in the present study), work ability, health-related QoL, pain, emotional functioning and their assessment methods, (5) research design, kind of study, study phase and follow-up time, and (6) statistical outcomes, conclusions and further statistical data. Data were extracted to a digital coding protocol by 2 reviewers (W.J.A.G., E.T.) independently, and compared for data accuracy and consensus before analysis.^34^

Descriptive analysis was then performed on this database. When coding was completed, all reported variables (potential prognostic factors), n≥200, were presented to the review team for a consensus-reaching grouping process, by which similar variables were collated into coherent domains, with related prognostic factors, to be used in further analyses. Variables that were too disparate to be included in any domain were specified in the original synthesis file, for transparency of the grouping process. When all found prognostic factors and domains were set, the analyses for the current study with the primary outcome physical functioning was initiated, parting the remaining outcome domains for later analysis.

A narrative synthesis of the relation between each potential prognostic factor and the outcome physical functioning was performed, in which the direction (positive, negative, or absence of association) was stated. Depending on how data were presented in the original studies, results were, if necessary, reversed to fit the chosen reporting direction of synthesis, that is for “positive outcome,” for example low levels of disability and high levels of physical functioning.

A quantitative synthesis was also performed. When at least 2 studies provided data on the same prognostic factor, a subsequent meta-analysis was aimed for, based on our a priori decision. All outcome data required for the meta-analyses were extracted from the coding protocol and complemented with details from the articles by the 2 reviewers together, and then double-checked once more. To quantify the strength of the relationship between identified prognostic factors and corresponding outcomes, the statistical outcomes (effect sizes) from single studies were converted into a common index to permit pooling across studies.^35^ The odds ratio (OR) was set as the common index used in our analyses, an effect size frequently used in prognostic studies. Web-based calculators^36^,^37^ were used to compute and transform any relevant data that were not reported as ORs, that is continuous and correlational data, into ORs and their 95% confidence intervals (CIs). The complete methodology for these procedures is descripted in Lipsey.^38^ In the software Review Manager,^39^ variance weighted pooled ORs were then computed in a random effects model for each prognostic factor, using the generic inverse variance method, which permits a wide selection of data formats in the analyses.^40^ For every meta-analysis, measures of statistical heterogeneity as expressed by τ^2^, χ^2^, and *I*^2^ were assessed. Funnel plots were used to assess potential publication bias, in accordance with our protocol, although the optimal number of studies was not reached.

In cases where *P*-values were reported as <0.05 or *, **, ***, instead of their exact value, the values were set as 0.049, 0.009, and 0.0009, respectively, and correspondingly, if presented as NS or >0.05, it was set as 0.051. During the syntheses, some authors were contacted for clarification or complementary data.^41^–^43^ Finally, we decided to exclude factors that were reported as dichotomous variables, because no continuous data were available and the resulting effect sizes became outliers in the meta-analyses. In studies with multiple comparisons or outcome measures within the same prognostic factor group, related data were first pooled into one estimate, to avoid double-counting and overestimation and then added to the meta-analysis.^44^

### Sensitivity and Moderator Analyses

Sensitivity was assessed for type of effect (fixed vs. random effects), study quality (including only studies with low RoB vs. including only studies with moderate/high RoB), follow-up time (studies with 6-mo follow-ups vs. studies with >6-mo follow-ups), and type of analysis (uni/multivariate) and measurement instruments when possible. The influence of every study on the effect size was assessed by excluding one at a time; the “leave one out” procedure.

### Evidence Synthesis

The quality of evidence for each reviewed potential prognostic factor was assessed using the GRADE method.^45^ Because the primary study type for high level of evidence (LoE) for prognosis is based on cohort study design, instead of the controlled experimental designs as preferred in inferential research of intervention effects, we followed the adapted framework as proposed by Huguet et al^46^ and Iorio et al^47^ to judge the quality of prognostic evidence. Here, evidence is evaluated by mainly the same factors, that is study limitations, inconsistency, indirectness, imprecision, and publication bias, but the phase of investigation plays a more important part, where explanatory studies of phase II and III constitute the starting point for a high LoE for prognosis.^46^ A 4-LoE was used: ++++ (high), indicating high confidence in that the true effect lies close to that of the estimate of the effect; +++ (moderate), indicating moderate confidence in the effect estimate; ++ (low), indicating limited confidence in the effect estimate, and + (very low), indicating very little confidence in the effect estimate.

The LoE was assessed independently by 2 reviewers (E.T. and W.J.A.G.) before consensus was reached. An overall judgment of the available data was made; from the coding protocol, the quality assessment, the narrative analyses, the meta-analyses, and the sensitivity analyses, and looked at the resulting compilation of studies for each prognostic factor. As recommended the initial evidence level was set upon the judgment of the *study phase*.^46^ If there was not a majority for low RoB, we downgraded for *study limitation*. Judgment of *inconsistency*, influencing the estimates of prognosis, was based on an evaluation of all analyses (narrative, quantitative, sensitivity, and the *I*^2^ statistics). *Indirectness*, generalizability, was assessed through an estimation of our included material. *Imprecision* was not deemed possible to judge in our study. *Publication bias* was assessed through funnel plots and a comparison of effects included in narrative and quantitative syntheses.

## RESULTS

### Results of the Literature Search

Electronic searches identified 3355 candidate studies, and 491 full text articles were retrieved. A total of 187 studies met the PICOT eligibility criteria and were subsequently screened once more for relevance. Of these, 105 studies met our relevance criteria and were included in the present review for further analysis, within the prespecified outcomes—physical functioning, pain, work, and QoL. During the data extraction and the process of narrative synthesis, additional studies were excluded for the following reasons: lack of sufficient data on the prognostic factors of interest (10), data provided only on change factors instead of baseline factors (10), mixed group analyses, that is prediction analyses of MDR-treated groups and control groups together (5), or double reporting of data (1), wrong outcome (1), or other (3). As a result, 75 studies remained eligible for analysis, and from these, the 25 studies that reported on prognostic factors for the outcome Physical functioning were selected for analysis and included in the present report (Fig. 1).

### Description of Studies Included in Analysis

The 25 included studies consisted of 24 cohort studies^41^–^43^,^48^–^68^ and 1 randomized controlled trial.^69^ Nineteen studies were primary prognostic factor studies while 3 examined prognosis as their secondary aim, and 2 examined validation/study methodology. Follow-up time ranged from 6 to 18 months with a loss to follow-up between 0% and 51% (median=14%). In total, 9436 participants were included in the studies, with sample sizes ranging from 39 to 3106 participants for the single studies (mean n=377, median n=143). The studies were published between 1983 and 2016. Nineteen studies were conducted in Europe (Sweden 6, Germany 4, the Netherlands 5, Norway 1, Finland 1, Denmark 1, Switzerland 1), 5 in North America (USA 3, Canada 2), and 1 in New Zealand (Table 1). Studies included patients with an average age ranging from 38 to 54 and the percentage of females in their samples ranged from 35% to 100%. Studied diagnoses were chronic (low) back pain (n=12), chronic pain (n=9), fibromyalgia (n=2), generalized widespread pain (n=1), and whiplash-associated disorder (n=1). The participants’ average pain duration ranged from 3 months to >10 years; the majority of them had had chronic (persistent) pain for several years. Participants were recruited or referred from primary care, secondary care, or insurance providers.

**TABLE 1:**
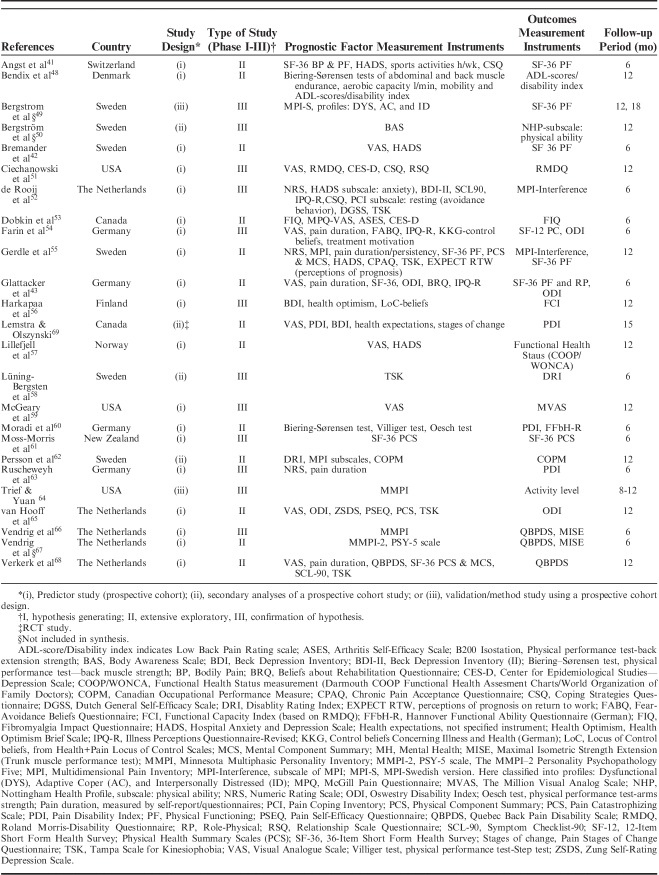
Description of Included Studies

Interventions were described using the following nomenclature; “multidisciplinary/multimodal/ interdisciplinary” (19), “functional restoration program” (5), and “work hardening program” (1). The intervention duration varied mainly between 2 and 8 weeks, although some interventions were performed in 2 phases, in which a longer follow-up period with continued rehabilitation time was offered for as long as a year. Twelve of 25 studies reported an MDR intervention time of 4 to 8 weeks, and 7 studies reported a longer duration; either >8 weeks or >8 weeks when both phases were added together. The majority of studies reported an average total of 100 hours, although this could be delivered as full time treatment over the period of a couple of weeks or more spread out over a couple of months (Table 2).

**TABLE 2:**
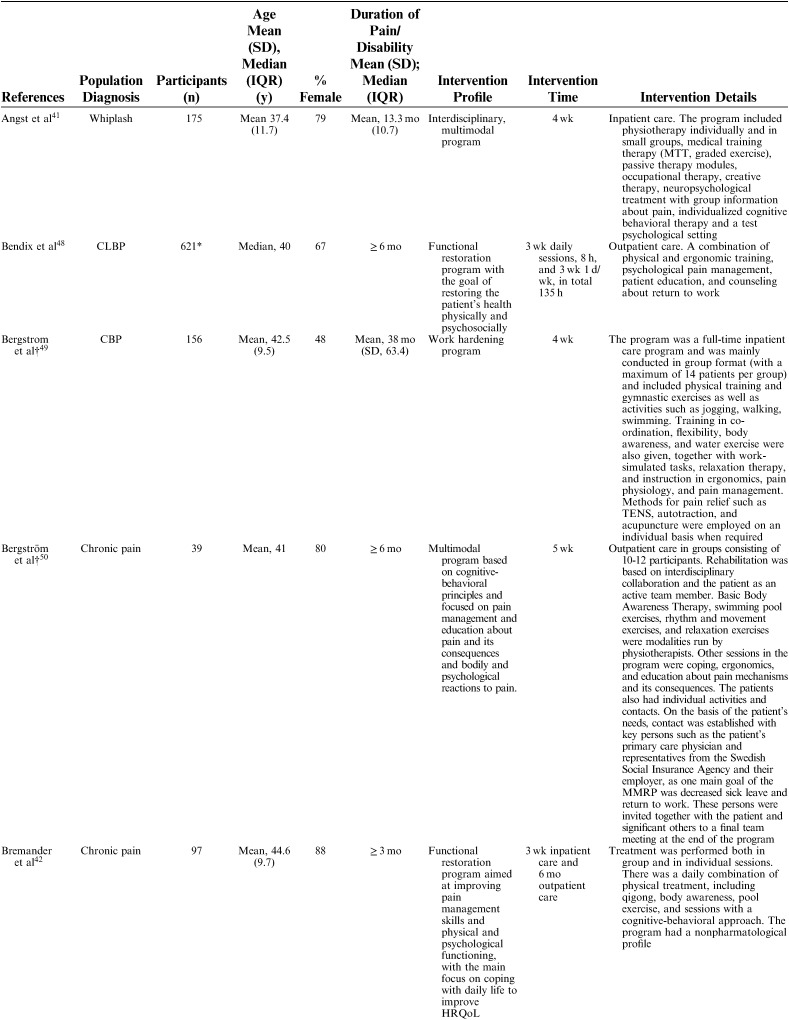
Description of Participants and Intervention

**TABLE 2:**
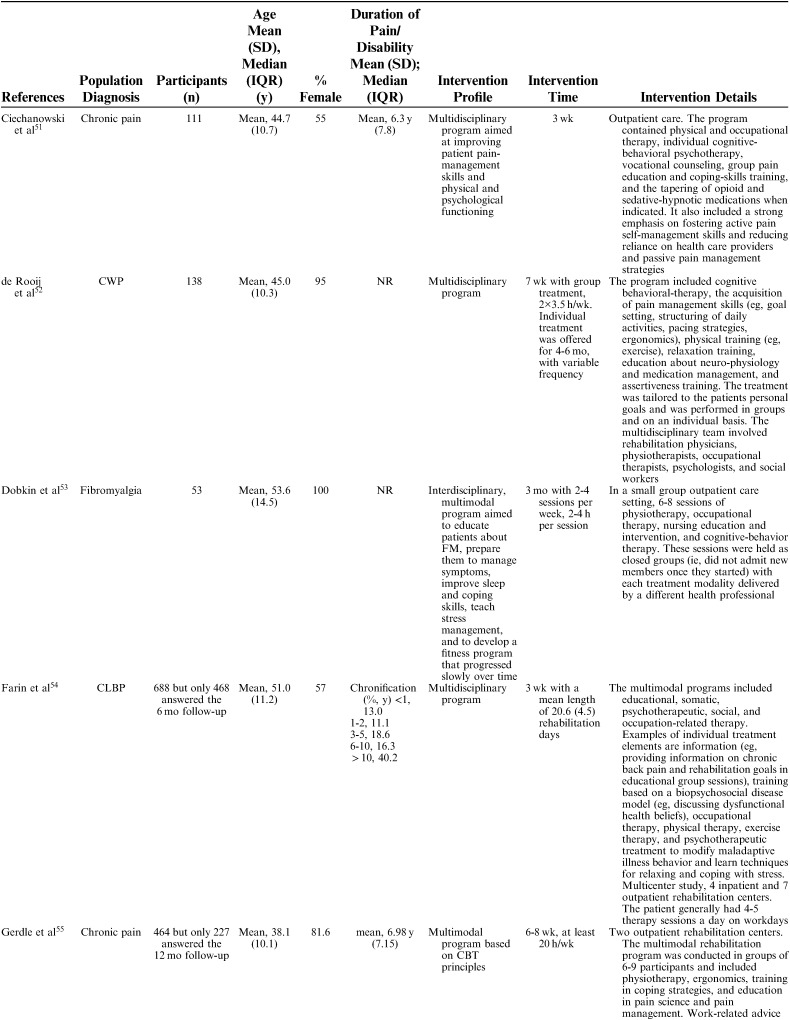
(continued)

**TABLE 2:**
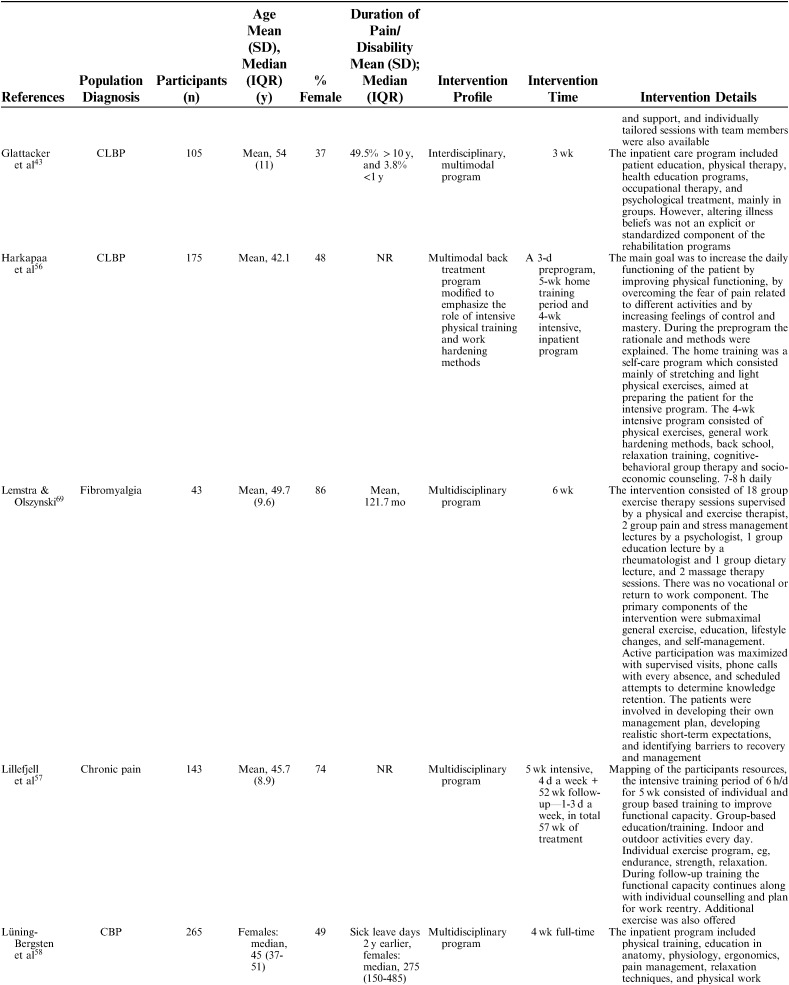
(continued)

**TABLE 2:**
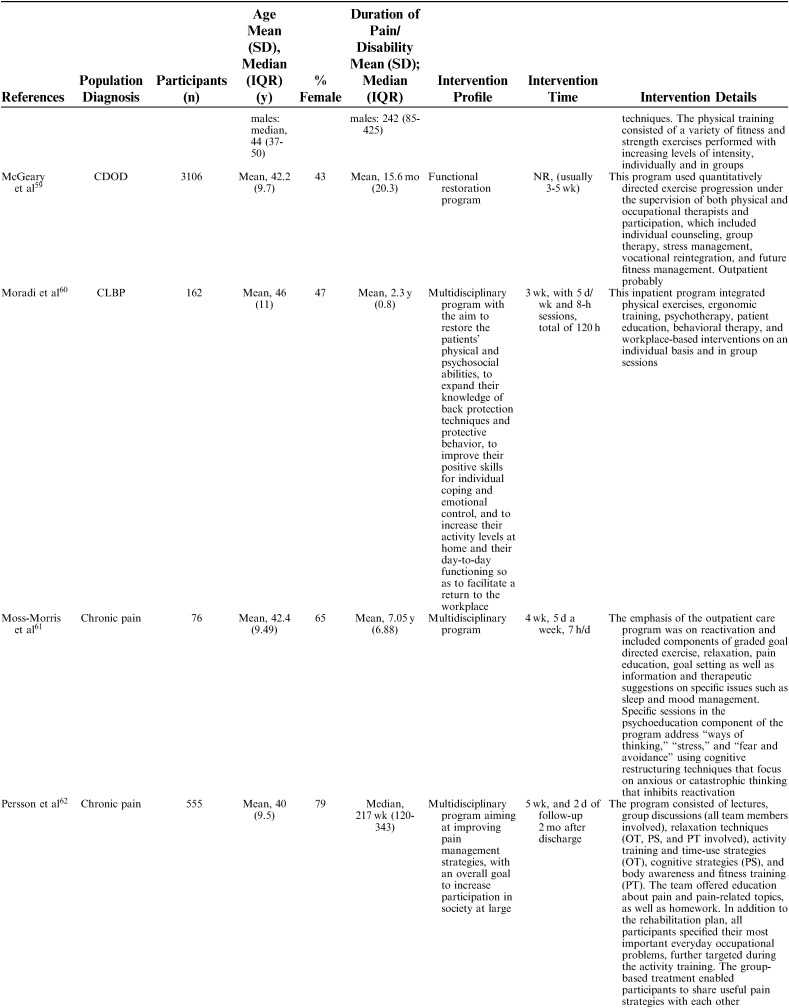
(continued)

**TABLE 2:**
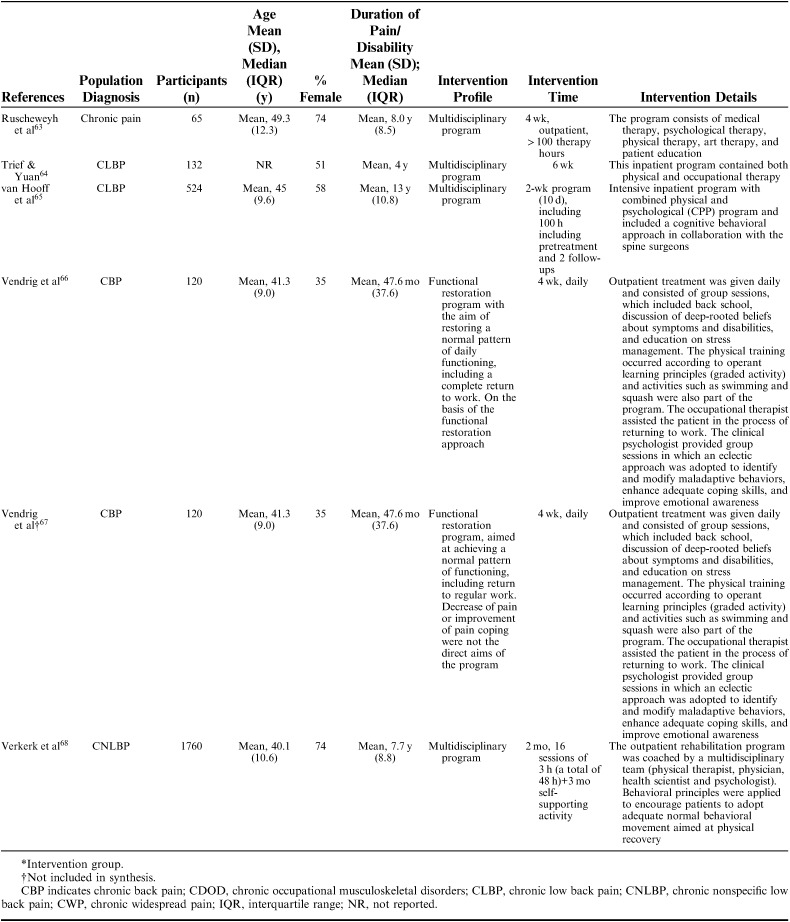
(continued)

### Outcome Measures

Both generic and disease-specific measures for physical functioning were used. The outcomes relating to physical functioning were assessed either with measures of physical functioning or measures of disability, or a combination of both. Outcome measures used to assess physical functioning, included ADL scores, the Coop Functional Health Assessment Charts (COOP/WONCA), Functional back capacity (FFbH-R), the Maximal Isometric Strength Extension (MISE), the Functional Capacity Index (FCI), and scales from the 36-Item Short Form Health Survey (SF-36); Physical Functioning (PF), Role-Physical (RP), Physical Component Summary (PCS) and respectively, the 12-Item Short Form Health Survey (SF-12). For disability, measures included the Roland-Morris Disability Questionnaire (RMDQ), the Oswestry Disability Inventory (ODI), the Disability Rating Index (DRI), the Quebec Back Pain Disability Scale (QBPDS), the Pain Disability Index (PDI), the Fibromyalgia Impact Questionnaire (FIQ), and the Multidimensional Pain Inventory (MPI)-Interference scale. Most of the measures were based on self-reports, that is Patient Reported Outcome Measures (PROM), whereas some were performance-based and assessed by the MDR team.

### Prognostic Factors

A total of 87 baseline factors were identified, which were operationalized into domains. Three domains and their related potential prognostic factors were included for synthesis; *Pain-related factors*, *Physical function-related factors*, and *Psychological factors*, in analogy to the assessment topics of the IMMPACT.

1. *Pain-related factors*: pain intensity and pain duration. Assessment measures included Numeric Rating Scale (NRS), Visual Analogue Scale (VAS), and the SF-36—Bodily pain (SF36-BP).
2. *Physical function-related factors*: performance-based function (e.g. muscle strength, mobility, aerobic capacity, and self-rated function, expressed in terms of physical ability or disability). Function-related factors were assessed with the same measures as the primary outcome (e.g., PDI, ODI, SF-36).
3. *Psychological factors*: psychological measures were sorted under higher order factors “emotional distress” and “cognitive-behavioral factors” to ensure relatively homogenous categories.^70^

•Emotional distress, for example, anxiety and depression. Assessment measures included the Hospital Anxiety and Depression Scale (HADS), the Beck Depression Inventory (BDI), the Center for Epidemiological Studies-Depression Scale (CES-D), the Minnesota Multiphasic Personality Inventory (MMPI), the Symptom Checklist-90 (SCL-90), and the SF-36 Mental component scale (SF-36 MCS).
•Cognitive-behavioral factors, either with a positive direction, for example, health optimism, personal control, and self-efficacy or with a negative direction, risk, for example, catastrophizing, fear of movement, avoidance behavior, and external locus of control. Assessment measures included the Health Optimism Scale, the Health Locus of Control Scale, the Dutch General Self-efficacy Scale (DGSS), Pain Self-Efficacy Questionnaire (PSEQ), the Pain Coping Inventory (PCI), the Coping Strategy Questionnaire (CSQ), the Tampa Scale of Kinesiophobia (TSK), the Revised Illness Perceptions Questionnaire (IPQ-R), the Beliefs about Rehabilitation Questionnaire (BRQ), the Minnesota Multiphasic Personality Inventory-2 (MMPI-2), the Multidimensional Pain Inventory (MPI), subscale Life control—some of which evaluate both risk and protective factors.

*Sociodemographic-related*, *Medical-related*, and *Work-related factors* were identified as well, but will be reported in a separate report, due to the large amount of diverse factors provided in these domains.

### Methodological Quality

**FIGURE 2:**
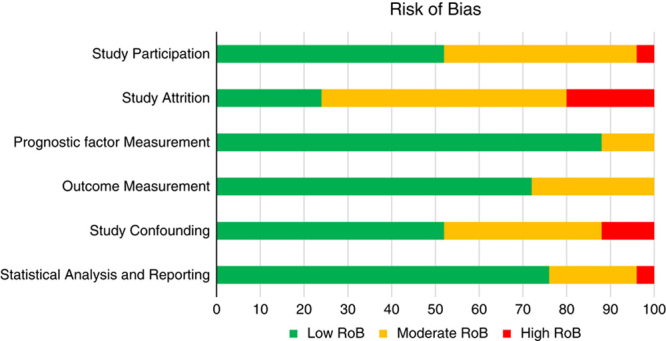
Risk of bias within studies as assessed in the 6 domains of the Quality in Prognostic Studies (QUIPS)-tool and presented as total percent of included studies (n=25).

The within-studies RoB is presented as a total percent of included studies for the 6 assessed domains of validity (Fig. 2). The domains study attrition and study confounding emerged with the highest RoB (ie, low quality), mainly due to insufficient reporting on these topics in the paper. Ratings for individual studies are displayed in Table 3.

**TABLE 3:**
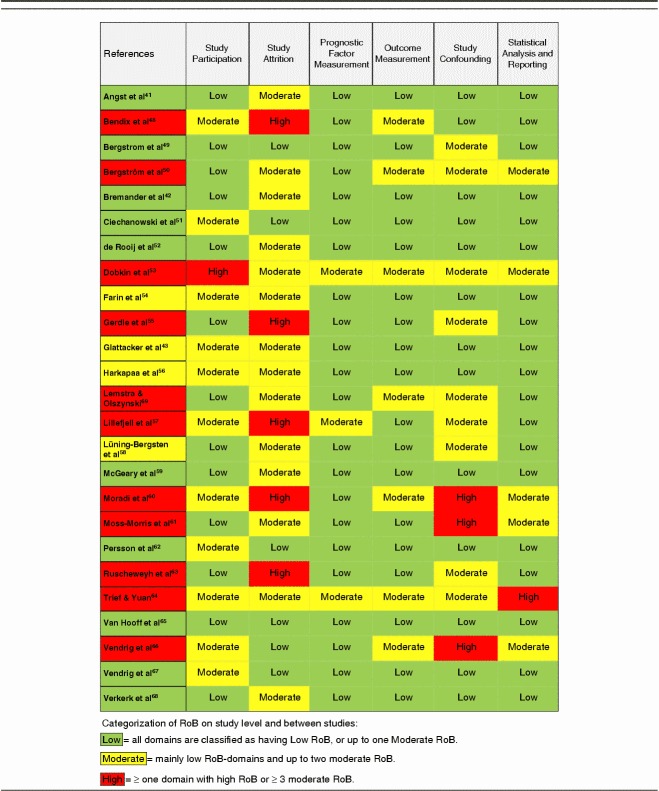
Risk of Bias (RoB) Ratings of the Included Studies, Assessed With the Quality in Prognostic Studies (QUIPS)-tool

## PROGNOSTIC FACTORS FOR PHYSICAL FUNCTIONING—NARRATIVE AND QUANTITATIVE ANALYSES

### Pain-related Factors

#### Pain Intensity

The association between baseline pain intensity and physical functioning after MDR was assessed in 16 studies,^41^–^43^,^48^,^51^–^55^,^57^,^59^,^62^,^63^,^65^,^68^,^69^ including a total of 8191 participants.

The narrative analyses indicated inconclusive results. Eight studies^42^,^43^,^52^,^53^,^55^,^63^,^68^,^69^ reported no association between pain intensity at baseline and outcome. Four studies^54^,^57^,^59^,^65^ showed that lower levels predicted positive outcomes while 2 studies^41^,^51^ showed that high pain levels at baseline predicted positive results at follow-up. Two studies had conflicting results, depending on pain location^48^ or type of analysis (uni/multivariate)^62^ (Table 4).

**TABLE 4:**
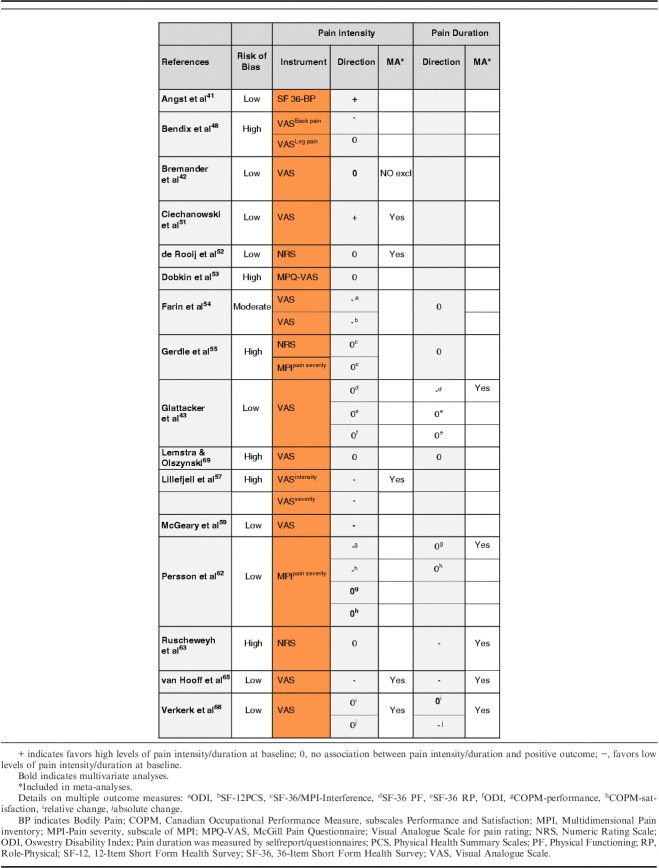
Narrative Analyses of Pain-related Factors

**FIGURE 3:**
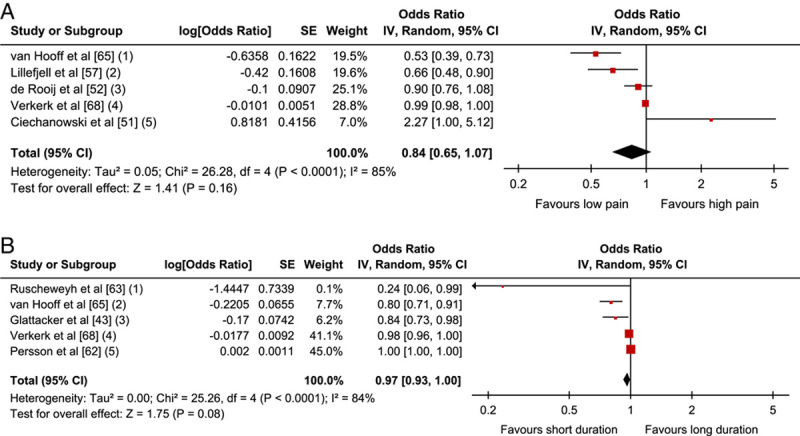
Pain-related factors: A, Forest plot showing baseline pain intensity and association with positive outcome. B, Forest plot of comparison between pain duration and association with positive outcome. The assessment measures for outcome and prognostic factor (PF) reported and type of analyses are presented in the footnotes. 3A; (1) Outcome: ODI; PF: NRS; univariate; (2) Outcome: COOP-WONCA; PF: VAS; multivariate; (3) Outcome: MPI interference; PF: NRS; univariate; (4) Outcome: QBPDS, relative and absolute recovery; PF: VAS; multivariate. Combined OR; (5) Outcome: RMDQ; PF: NRS average pain intensity past week; zero-order correlations. 3B; (1) Outcome: PDI; PF: pain duration, self reported; (2) Outcome: ODI; PF: pain duration, self reported; (3) Outcome: SF-36 PF; PF: pain duration 0-5 years; multivariate; (4) Outcome: QBPDS, absolute change; PF: pain duration, self reported; (5) Outcome: COPM >2 change; PF: pain duration, self reported. CI indicates confidence interval; COPM, Canadian Occupational Performance Measure; ODI, Oswestry Disability Index; OR, odds ratio; PDI, Pain Disability Index; PF, Physical Functioning; QBPDS, Quebec Back Pain Disability Scale; SF-36, 36-Item Short Form Health Survey.

Five studies (4 low, 1 high RoB) provided continuous data for inclusion in a meta-analysis (n=2676). Results of the meta-analysis showed that initial pain intensity was not associated with improvement in physical function at follow-up, OR=0.84; 95% CI, 0.65-1.07; *P*=0.16 (Fig. 3A).

#### Pain Duration

The association between pain duration before MDR and physical functioning was assessed in 8 studies^43^,^54^,^55^,^62^,^63^,^65^,^68^,^69^ including a total of 3800 participants.

Four of 8 studies^54^,^55^,^62^,^69^ reported no association with outcome, 2 studies showed a negative association,^63^,^65^ and 2 studies^43^,^68^ reported conflicting results on multiple outcome measures, showing either no association or a negative association in favor of short duration (Table 4).

Five studies (3 low, 1 moderate, 1 high RoB) were included in a meta-analysis (n=2978). The pooled OR (95% CI) showed no association with physical functioning; that is, the results indicate pain duration at baseline is not a prognostic indicator for outcome, OR=0.97; 95% CI, 0.93-1.00; *P*=0.08 (Fig. 3B).

#### Sensitivity Analyses and LoE (GRADE)

The sensitivity analyses for both pain intensity and pain duration showed that our results remained robust when examining the influence of study quality, follow-up time, measurement instruments, uni/multivariate analyses, and when compared with a fixed-effects model. The GRADE analyses of pain intensity as well as pain duration showed that, due to downgrading as a result of “inconsistency of the results,” there is evidence of moderate quality that baseline pain level and pain duration cannot predict physical functioning at ≥6-month follow-up of MDR (Table 7).

### Physical Function–related Factors

The association between baseline and follow-up physical functioning was assessed in 15 studies (n=4868).^41^,^43^,^48^,^51^–^53^,^55^–^57^,^60^–^62^,^65^,^68^,^69^ Physical function was assessed either by patients’ actual performance of physical tests (and evaluated by therapists)—or by patients’ own reporting of their function, activities, or disability, that is completing questionnaires (PROMs). The factors were divided into 2 groups and analyzed separately due to the qualitative differences of the assessment methods (Table 5).

**TABLE 5:**
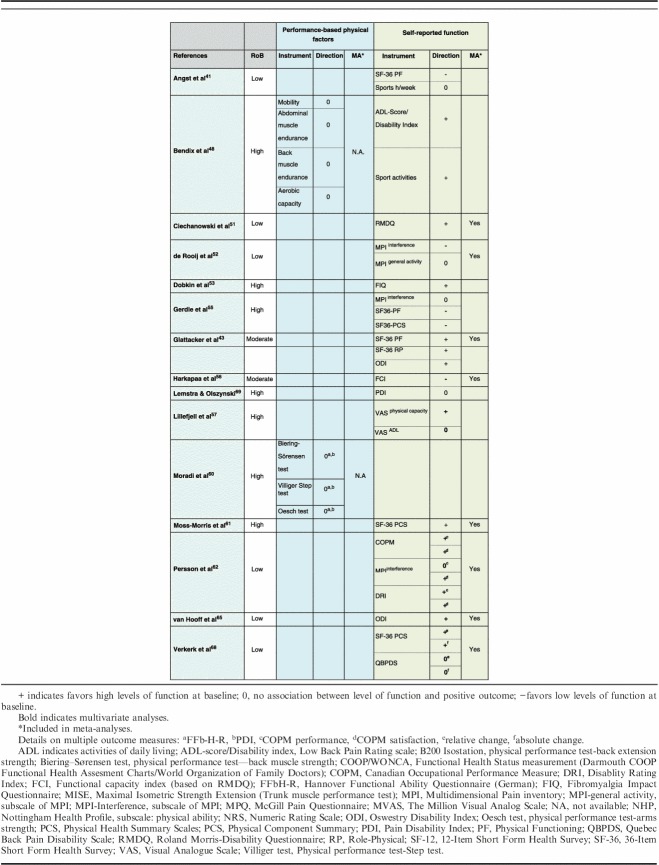
Narrative Analyses of Physical Function-related Factors

#### Performance-based Physical Factors

Two studies^48^,^60^ investigated 6 performance-based physical factors (n=783). The tests evaluated isometric endurance, mobility, and aerobic capacity as prognostic factors. The narrative analyses indicated no prognostic value for outcomes related to physical function, both studies reported no significant association. Both studies were rated as having high RoB. Because of limited data, a meta-analysis was not appropriate.

#### Self-reported Function, Activities/Disability

Fourteen studies examined the association between self-reported physical functioning and outcome (n=4706).^41^,^43^,^48^,^51^–^53^,^55^–^57^,^61^,^62^,^65^,^68^,^69^

The narrative analyses of self-assessed physical function revealed inconclusive results. Higher levels of function at baseline were significantly associated with a positive outcome in 6 studies, while low levels of function associated with a positive outcome were reported in one study and no significant association was reported in another one study. However, 6 studies presented inconclusive results depending on measures used, either showing an inconsistency between a positive association and no association (3 studies) or between a negative association and no association (3 studies).

**FIGURE 4:**
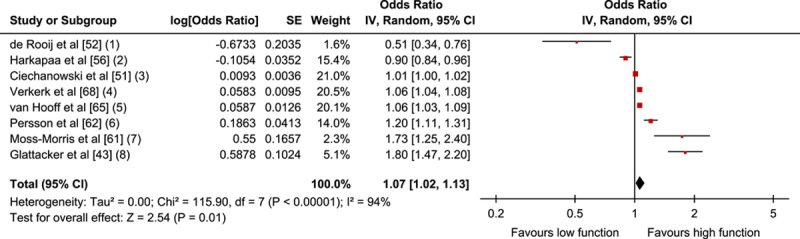
Physical Function-related factors: Forest plot of comparison between baseline function and association with positive outcome. The assessment measures for outcome and prognostic factor (PF) reported, type of analyses, and whether estimates (ORs) were combined from plural measures are presented in the footnotes. Physical function (1) Outcome: MPI interference; PF: MPI interference, MPI activity level; uni & multivariate. Combined OR; (2) Outcome: FCI; PF: FCI, univariate; (3) Outcome: RMDQ; PF: RMDQ; univariate zero-order correlations; (4) Outcomes: QBPDS absolute and relative change; PF: SF-36 PCS; multivariate. Combined OR; (5) Outcome: ODI; PF: ODI; multivariate; (6) Outcomes: COPM satisfaction and performance; PF: COPM satisfaction, performance and DRI; multivariate. Combined OR; (7) Outcome: SF-36 PCS; PF: SF-36 PCS; univariate; (8) Outcomes: ODI, SF-36 PF and SF-36 RP; PF: ODI, SF-36 PF, SF-36 RP; multivariate. Combined OR. CI indicates confidence interval; COPM, Canadian Occupational Performance Measure; MMPI, Minnesota Multiphasic Personality Inventory; MPI, Multidimensional Pain Inventory; ODI, Oswestry Disability Index; OR, odds ratio; PF, Physical Functioning; QBPDS, Quebec Back Pain Disability Scale; RP, Role-Physical; SCL-90, Symptom Checklist-90; SF-36, 36-Item Short Form Health Survey.

Eight studies (5 low, 2 moderate, 1 high RoB) were included in a meta-analysis (n=3444). The pooled OR (95% CI) showed that high baseline function was associated with positive outcome, OR=1.07; 95% CI, 1.02-1.13; *P*=0.01 (Fig. 4).

#### Sensitivity Analyses and LoE (GRADE)

The results of self-reported physical function remained robust when excluding high RoB studies, and were independent of a fixed or random model. However, when analyzing the 3 studies^43^,^52^,^61^ with shorter follow-up times, there was no longer any significant association between physical function at baseline and outcome. Moreover, in studies with univariate analysis only,^51^,^56^,^61^ the associations disappeared as well.

The Grade synthesis showed there was no evidence (−) of prognostic value of performance-based physical function and that there was low evidence (++) of a small effect of self-rated initial high physical functioning as prognostic for good physical functioning at follow-up after MDR (Table 7). Downgrading was due to “study limitations” and “inconsistency of the results.” For performance-based physical function, the initial GRADE LoE was set at +++, due to unclear study phases.

### Psychological Factors

Seventeen studies^41^–^43^,^49^,^51^,^52^,^54^,^56^–^58^,^62^,^64^–^69^ investigated baseline psychological factors. Of these, most were categorized as either emotional factors or cognitive behavioral factors. For the purpose of analyses, cognitive-behavioral factors were divided into protective factors or risk factors. A few remaining factors, mostly relating to personality traits,^51^,^64^,^67^ were considered too compound or dissimilar and were therefore not synthesized in this context.

#### Emotional Factors

Fifteen studies (n=4358)^41^–^43^,^51^–^53^,^55^–^57^,^62^,^64^–^66^,^68^,^69^ investigated emotional factors relating to mood/distress, for example, depression and anxiety and their association to physical functioning at follow-up.

The narrative analyses showed inconclusive results concerning their prognostic value. Six studies^43^,^51^,^53^,^55^,^56^,^69^ did not demonstrate any significant associations, 6 studies^41^,^52^,^62^,^64^,^66^,^68^ showed differing results between anxiety and depression, 2 studies^57^,^65^ showed that low levels of depression/anxiety at baseline could predict positive results at follow-up, while 1 study^42^ showed some degree of initial anxiety/depression was associated with a positive outcome. Anxiety and depression were analyzed both separately and in combination with each other (Table 6).

**TABLE 6:**
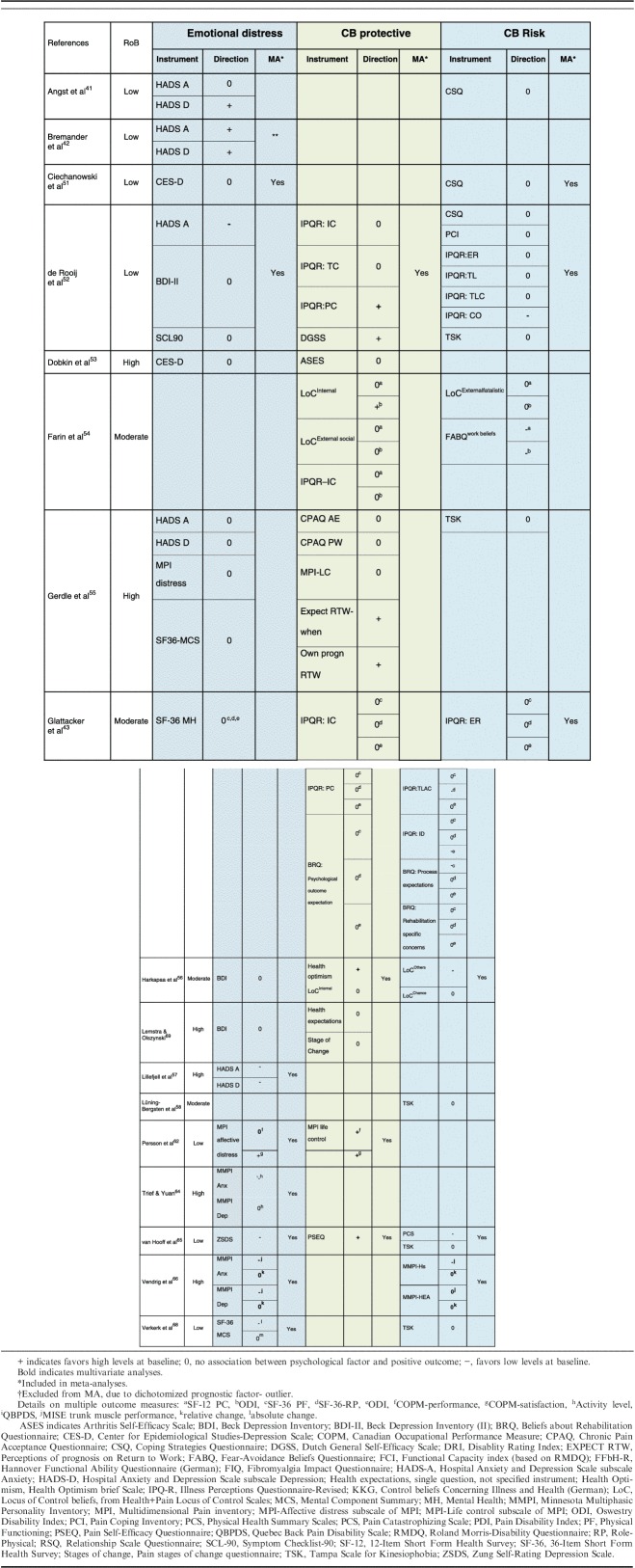
Narrative Analyses of Psychological Factors

**FIGURE 5:**
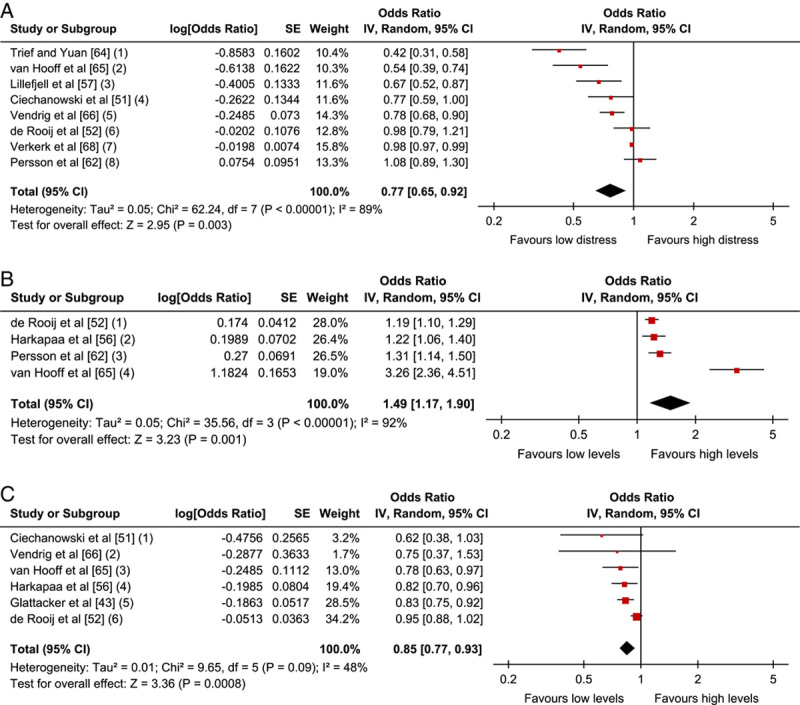
Psychological factors: A, Forest plot of comparison between baseline emotional distress and association with positive outcome. B, Forest plot of comparison between baseline levels of cognitive behavioral protective factors and association with positive outcome. C, Forest plot of comparison between baseline levels of cognitive behavioral risk factors and association with positive outcome. The assessment measures for outcome and prognostic factor (PF) reported, type of analyses, and whether estimates (ORs) were combined from plural measures are presented in the footnotes. 5A; (1) Outcome: Activity level; PF: MMPI-Anx Pt; univariate; (2) Outcome: ODI; PF: Zung Self-rated Depression scale; univariate; (3) Outcome: COOP-WONCA; PF: HADS-A and HADS-D; multivariate. Combined OR; (4) Outcome: RMDQ; PF: CES-D; univariate correlation; (5) Outcomes: QBPDS and MISE; PF: Anxiety: MMPI-2 Pt, ANX and PBS and Depression: MMPI-2 D and DEP; multivariate. Combined OR; (6) Outcome: MPI Interference; PF: HADS-A and SCL-90 psychological functioning; multivariate and BDI-II, univariate. Combined OR; (7) Outcomes: QBPDS, relative and absolute recovery; PF: SF-36MCS; multivariate. Combined OR; (8) Outcome: COPM satisfaction; PF: MPI Affective distress: multivariate. 5B; (1) Outcome: MPI Interference; PF: DGSS, Illness coherence, IPQ Personal control, IPQ Treatment control; uni- and multiv. Combined OR; (2) Outcome: FCI; PF: Health optimism; multivariate; (3) Outcomes: COPM Performance and Satisfaction; PF: MPI Life control; multivariate. Combined OR; (4) Outcome: ODI; PF: PSEQ self-efficacy; univariate. 5C; (1) Outcome: RMDQ; PF: CSQ; univariate; (2) Outcomes: QBPDS, MISE; PF: MMPI-2 Hs, MMPI-2 HEA; multivariate. Combined OR; (3) Outcome: ODI; PF: TSK and PCS. Combined OR; (4) Outcome: FCI; PF: Other LoC; multivariate; (5) Outcome: ODI and SF-RP; PF: IPQ-R timeline acute-chronic, BRQ identity, BRQ process expectation; multivariate. Combined OR; (6) Outcome: MPI Interference; PF: IPQ-R; Timeline, Conseq., Emotional repr., Timeline cycl., PSQ, PCS, TSK; uni-and multiv. Combined OR. BDI indicates Beck Depression Inventory; CI, confidence interval; COPM, Canadian Occupational Performance Measure; COOP/WONCA, Coop Functional Health Assessment Charts HADS, Hospital Anxiety and Depression Scale; MISE, Maximal Isometric Strength Extension; MPI, Multidimensional Pain Inventory; ODI, Oswestry Disability Index; OR, odds ratio; PF, Physical Functioning; QBPDS, Quebec Back Pain Disability Scale; RMDQ, Roland-Morris Disability Questionnaire; RP, Role-Physical; SF-36, 36-Item Short Form Health Survey.

Eight studies (5 low, 3 high RoB) with continuous data were included in a meta-analysis (n=3483). The pooled OR (95% CI) showed that there was a small, statistically significant, association between low baseline emotional distress and a positive outcome, OR=0.77; 95% CI, 0.65-0.92; *P*=0.003 (Fig. 5A).

#### Cognitive and Behavioral Factors—Protective Factors

Nine studies (n=2288)^43^,^52^–^56^,^62^,^65^,^69^ examined various cognitive and behavioral factors relating to self-efficacy, control beliefs, and health optimism; factors commonly attributed to strengthening a person’s resilience, that is with a protective effect.

The narrative analyses showed diverse results. Three studies^43^,^56^,^69^ found no association from 6 examined protective factors, while 3 studies showed a positive association favoring high levels of 3 identified protective factors^56^,^62^,^65^ and 1 study^43^ showed a negative association, indicating low levels of 1 factor was associated with a positive outcome.

Four studies (3 low, 1 moderate RoB) were included in a meta-analysis (n=1392). The pooled OR (95% CI) showed, contrary to the narrative analysis, an association between high levels of protective cognitive behavioral factors and a positive outcome, OR=1.49; 95% CI, 1.17-1.90; *P*=0.001 (Fig. 5B).

#### Cognitive and Behavioral Factors—Risk Factors

Eleven studies (n=4068)^41^,^43^,^51^,^52^,^54^–^56^,^58^,^65^,^66^,^68^ examined the association between various “negative” cognitive and behavioral factors and outcome, that is potential risk factors. These were related to illness and self-efficacy beliefs, fear-avoidance beliefs and behavior, catastrophizing, and dimensions of somatic discomfort/somatization.

The narrative analyses of cognitive and behavioral risk factors indicated a majority of nonsignificant associations. Results identified 20 items with no association and 9 in favor of low levels for a positive outcome.

Six studies (2 low, 3 moderate, and 1 high RoB) were included in a meta-analysis (n=1173). The pooled OR (95% CI) showed, contrary to the narrative analysis, an association between low levels of cognitive and behavioral risk factors and a positive outcome, OR 0.85; 95% CI, 0.77-0.93; *P*=0.0008 (Fig. 5C).

#### Sensitivity Analyses and LoE (GRADE)

Sensitivity analyses of *emotional* factors showed that the significant associations disappeared when including only studies with low RoB and the OR increased from 0.77 (95% CI, 0.65-0.92) to 0.89 (95% CI, 0.75-1.04) and to 0.90 (95% CI, 0.78-1.03) when only including studies with multivariate analyses. In addition, when only the 2 studies with a 6-month follow-up were included, the association disappeared (OR, 0.86; 95% CI, 0.69-1.08). However, the results remained robust when comparing anxiety/depression separately and when compared with a fixed effects model.

The results remained robust through all sensitivity analyses of *protective* factors; study quality, follow-up time, univariate/multivariate data and when compared with a fixed effects model. The OR increased from 1.49 (95% CI, 1.17-1.90) to 1.67 (95% CI, 1.12-2.49), when including studies with follow-up periods of longer than 6 months.

Sensitivity analyses of *risk* factors showed that the significant associations disappeared when including only studies with low RoB or studies with short follow-up time (6 mo). However, the OR changed by <0.06 and the results remained robust when comparing univariate/multivariate data and when compared with a fixed effects model. All in all, sensitivity analyses of the psychological factors clearly showed that the results were robust.

In summary, based on a GRADE analysis of these results including sensitivity analyses, the results showed that (a) there is moderate quality evidence that low initial emotional distress predicts a positive outcome on physical functioning at follow-up after MDR, (b) there is moderate quality evidence that high levels of protective cognitive behavioral factors predict a positive outcome of physical functioning at follow-up after MDR, and (c) there is moderate quality evidence that low levels of cognitive behavioral risk factors predict a positive outcome (Table 7). Downgrading was due to “study limitations” (a, c) and suspected “publication bias” (b).

**TABLE 7:**
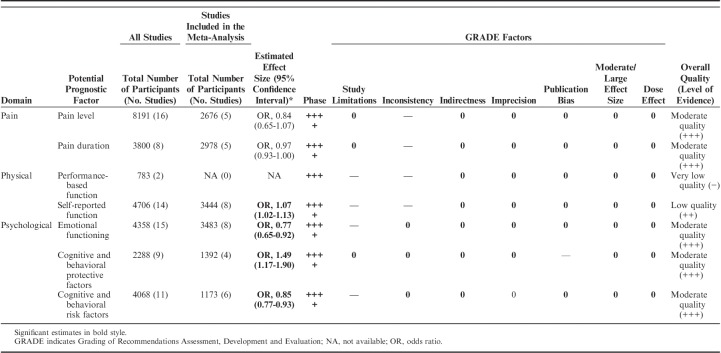
Summary of Findings and Overall Quality as Assessed With GRADE

## DISCUSSION

### Summary of the Results

To synthesize the evidence on prognostic factors for long-term (≥6 mo) physical functioning in patients with chronic musculoskeletal pain after MDR treatment, we examined 25 studies (n=9436) that included a total of 87 potential prognostic factors relating to initial pain and physical and psychological functioning.

The key finding of this review confirmed that pretreatment psychological factors as well as physical function/disability are important prognostic indicators of functional outcome after MDR while common pain variables did not appear to provide evidence on prognosis.

Regarding psychological factors, results showed a moderate LoE that low levels of emotional distress, high levels of cognitive and behavioral protective factors, and low levels of cognitive and behavioral risk factors predicted a better physical functioning in long-term follow-up. Moreover, results showed a low LoE that high levels of self-reported physical function predicted better physical functioning. Our results also indicated, with moderate levels of evidence, that pain severity and pain duration did not predict physical functioning after MDR in patients with chronic musculoskeletal pain at least 6 mo after treatment.

### Comparison With Previous Reviews

#### Pain Factors

Our study found that pain severity and pain duration did not have any prognostic value (moderate LoE), indicating that pretreatment information on pain per se is not informative for the further clinical course, at least not where physical function is concerned. The review of van der Hulst et al^20^ also reported that pain duration lacked prognostic value. But contrary to our study, they found evidence that higher pain intensity was associated with worse outcome. However, this conclusion was based on only 2 articles, one of which is included in our study,^48^ while the other study included findings on a dissimilar subgroup of population, intervention, and outcome. On the other hand, the review of de Rooij et al^19^ reported the opposite, that is high pain intensity being associated with a better outcome, though this conclusion was based on only 1 study. In previous reviews^22^,^24^ that have investigated prognostic ability in earlier phases of pain chronicity (acute and subacute), pain variables presented with evidence of a negative impact on outcome. In our results, however, pain ratings were not significantly related to the outcome, in this case physical functioning, although the direction of the association was in accordance to these previous results, maybe indicating a less prognostic value over time.

#### Physical Factors

In the synthesis we differentiated between objectively measured performance-based and self-assessed physical functioning. The assessment of performance-based function was only investigated in 2 studies, and showed no association, which is in line with van der Hulst et al.^20^ Moreover, the study of Wessels et al,^21^ which investigated the association of *changes* in physical performance factors with improvement in disability, also reported that there was no association with outcome. Further research is needed to elucidate the topic, to investigate whether more objectively measured dimensions of physical functioning could have a prognostic value for outcome. On the other hand, self-assessed physical functioning emerged as a major outcome topic, and proved valuable in predicting outcome. We found, with low levels of evidence, that self-assessed physical function predicts physical functioning 6 mo after MDR. Our meta-analysis strengthened the results from the qualitative analyses of van der Hulst et al,^20^ where it was found that self-assessed physical functioning could predict physical functioning. Also, as the findings were reproduced in a mixed-diagnosis chronic pain population—instead of a more homogenous chronic low back pain population—the generalizability of the findings increased. However, the reasons for the inconsistency in reported direction of the association (either favoring higher or lower baseline status), which were also noted by van der Hulst and colleagues, need to be further examined.

#### Psychological Factors

We found high levels of emotional distress predicted poor outcome, which is in line with previous assumptions and reports^19^,^70^,^71^; however, there is a lack of consistent evidence.^20^ This is the first time it has been shown in a meta-analysis based on >3000 participants, and our results confirm the importance of patients’ emotional functioning for treatment outcome.

Cognitive and behavioral factors are implied to have an impact on treatment outcome^19^,^20^,^70^ and this was also confirmed by our results. These essential factors of the pain experience may both strengthen the ability to deal with chronic pain as well as hinder patients’ adaptation. The narrative analyses of cognitive behavioral risk factors indicated a majority of nonsignificant associations but the meta-analysis revealed them to be significant prognostic factors for a negative outcome. While addressing these factors is at the core of pain management in MDR, our results show that high levels on cognitive and behavioral risk factors are related to poorer functional outcome. This implies that our current best evidence practice may not be addressing the coping problems of these patients satisfactorily. Indeed, Morley et al^72^ pointed out that results of cognitive-behavioral therapy pain management programs are modest at best, and these results have led to calls for improvements in treatment models.^73^,^74^

High levels of cognitive and behavioral protective factors predicted a better level of physical functioning in long-term follow-up. The results confirm the importance of factors attributed to a person’s resilience in determining outcome. Indeed, in a recent publication, the importance of factors related to a positive affect has been lifted forward as one way to improve treatments for chronic pain.^75^ As psychological risk and protective cognitive and behavioral factors are not mutually exclusive, MDR treatment should focus on both lowering the psychological risk factors and enhancing the protective psychological factors.

The prognostic ability of the psychological factors with a negative bearing, emotional distress (OR=0.77), and cognitive and behavioral risk factors (OR=0.85), respectively, was somewhat lower compared with the prognostic ability of the psychological protective factors (OR=1.49). This could be due to treatment effects, as in most MDR treatment programs the negative psychological factors are often targeted, while protective psychological factors may not be as commonly addressed. As previously put forward by de Rooij et al,^52^ prognostic factors that are targeted and altered during treatment can lose their prognostic ability, which may also be reflected in the present results. This could point to a more active clinical use of these positive, psychological protective factors for prognosis.

On the whole, as today’s management of chronic pain still gains only moderate effects, and the evidence to guide optimal treatment tailoring is limited, the importance of identifying prognostic indicators is of major clinical relevance. A prerequisite is that we are able to identify who is at risk of poor outcomes and who is most likely to benefit. Until now, no previous meta-analysis review studies have been conducted on this topic and, to our knowledge, this study is the first well-powered systematic review to summarize the available literature on prognostic factors specifically for this major patient group.

### Methodological Considerations

The strength of this systematic review is that it synthesizes factors of importance for physical functioning, one of the main targeted outcomes of MDR, rather than exploring a single prognostic factor impact or a selected part of the chronic pain-population, for example based on diagnosis. The study takes its standing point from a pragmatic perspective, hypothesizing that some factors probably exist that are common for the chronic pain population in general, irrespective of initial pain diagnosis, that is generic factors of importance for treatment outcome. From a methodological point of view, a body of evidence derived from longitudinal and pragmatic cohort studies enables high confidence in the field of prognosis, in comparison to more selected experimental randomized controlled trial studies.^47^ On the other hand, attrition and confounding can limit the internal validity of observational studies. The way of creating high-level evidence by unifying these observational studies with systematic synthesis methods is therefore a strength of this study.

The interdisciplinary review team with expertise in all fields relating to the aim of the study enabled a precise study selection, which led to great confidence in the identification of both the population of interest and the intervention of interest. The team was generally in agreement during the study selection process, despite the heterogeneity of retrieved studies. Good inter-rater agreement was strived for in all selection steps and RoB ratings, by introducing every phase with a pilot.

Omitting gray literature is likely to have introduced some information bias; however, it would be too time consuming to also collect and deal with this type of spread-out information, which is often not reported in enough detail. Including only articles in English is a potential source for information bias as well; however, it was a necessity for maintaining the strictness and specificity during the scrutiny of the study selection process. In addition, some reporting biases, for example publication bias or selective reporting of outcomes or analyses, cannot be ruled out. Significant results have a greater chance of being made available. Still, we found many studies presenting nonsignificant results. We believe this was partly due to our broad review scope and an exploratory search strategy, which permitted a vast amount of material, independent of primarily targeted prognostic factors in the original research publications. We put great effort into using these, often nonsignificant, variables in our syntheses, either narratively or quantitatively if data were provided. This has hopefully led to adding power and reducing possible asymmetry. As the relatively small number of studies reporting on each comparison precluded a detailed and meaningful analysis of funnel plots for publication bias, we attempted to visually analyze the narrative tables for symmetry of significant versus nonsignificant reporting. For some results, for example, the synthesis of protective psychological factors, the effect emerged stronger in the meta-analysis, which could likely be a result of missing nonsignificant data.

The risk of selection bias may have been introduced in the initial screening of titles, which was performed by one reviewer instead of 2. However, it was necessary to reduce the recall volume resulting from the broad and sensitive search strategy—and this stage therefore dealt only with identifying titles that precluded inclusion. The following screening process had a robust arrangement with randomization of studies and independent teams constituted by a senior and junior researcher.

Other sources for limitations of the study results may arise if narrative and quantitative syntheses are based on incompatible study heterogeneity or low study quality. We aimed to provide a well-powered overview of potential prognostic indicators of various MDR outcomes—as a result heterogenous studies were included with regard to types of pain conditions/regions and clinical settings. This was based on the premise that common prognostic factors for “the chronic pain disease itself” probably exist. Although unique in its kind, some loss of specificity is therefore a consequence and limit to this review. To the best of our ability, great effort was put into a sensible study selection and a coherent collating of our found predictors and outcomes, in the sense of minimizing incompatible (noncomparable) factors. We are thus confident that the study populations and study interventions constituted a sample in accordance with the pragmatic, wide selection of individuals with chronic pain that would normally participate in MDR. The same applies for the grouping of factors and outcomes, which were measured with various instruments; however, all with the intention of capturing dimensions of the same construct. Incompatible measures or measures with measurement properties considered to be too vague were not included in analyses. In the present study, the OR was used as the common index in the meta-analysis, although the OR has sometimes been criticized for its difficulty in interpretation. We stated in our study protocol that we will present associations between prognostic factors and outcome by means of OR, and this could enhance comparisons with future MAs.^25^ A random effects model was chosen for the statistical analyses, as it assumes and deals better with the anticipated heterogeneity.

Heterogeneity, measured by *I*^2^, was generally high for almost all comparisons (range: 48% to 94%). Although *I*^2^ indicated high heterogeneity, our attempts to investigate the source for these differences did not reveal any systematic reasons for the variance. Sensitivity analyses proved our results were in general robust. The direction of the associations remained stable and did not result in any major change of variation in the effect, except for the factor physical functioning. The effect estimates remained stable when comparing studies based on statistical analyses (univariate vs. multivariate) and study quality (low vs. high), and follow-up time (shorter vs. longer), although the statistical significance level occasionally decreased to nonsignificant for the emotional distress and cognitive and behavioral risk factors. Sometimes the effects of the prognostic factors seemed to be strengthened over time, when comparing shorter versus longer follow-up time (eg protective cognitive and behavioral factors), but the limited number of included studies in each meta-analysis did not permit further detailed moderator analyses of follow-up time or further aspects of clinical diversity.

Although our sensitivity analysis of potential factors influencing the stability of our results was generally stable, we cannot exclude true heterogeneity. With more unexplained variance across studies, some caution in the interpretation of the results was required and we therefore downgraded all pain and physical function domains in the GRADE, due to “inconsistency.”

Study quality, that is poor methodological quality may also impose limitations to the validity of study results, for example, low power, low attrition rates, or inadequate analyses are likely to affect the estimates and widen the 95% CIs in smaller studies. Our included studies were to a large extent of good methodological quality, with at least two thirds having low or moderate RoB. Still, “study limitations” was the most common reason for downgrading the GRADE. The measures for both outcomes and prognostic factors were mainly of “good” quality and statistical analyses were relevant but attrition and dealing with confounding were the weakest domains—which can seriously impact the results in prognostic factor studies. The assessment of study quality relies to a great extent to the level of relevant reporting. Often study quality was downgraded due to unclear detailing on, for example, study participation and attrition, which might not have been actual sources for bias. Moreover, for some RoB domains, the PABAK-OS was found to be unacceptably low. However, it was easy to obtain consensus on the overall RoB scores during the consensus discussions. On a general note, it was apparent that reporting has improved over the past decades, possibly as a result of the devise of reporting guidelines, for example the STROBE checklist. All in all, we believe our results have external validity and can be generalized within the context of the population and intervention of interest—still keeping in mind that our findings may apply to this specific outcome “Physical functioning” and possibly not to the other dependent variables that will be analyzed in subsequent reviews.

## CONCLUSIONS

Physical functioning at 6 months or longer after MDR was not predicted by initial pain level or pain duration (chronicity), contrary to previous indications, and therefore should not be used for assumptions of treatment prognosis. Better physical functioning was predicted by high levels of initial self-assessed physical functioning. Furthermore, a better outcome was predicted by low levels of emotional distress and low levels of cognitive and behavioral risk factors, indicating that treatment should further target and optimize these modifiable factors. Finally, high levels of protective cognitive and behavioral factors were strong prognostic indicators of better physical functioning at 6 months or more after MDR, and an increased focus on positive, psychological protective factors may perhaps provide an opening for yet untapped clinical gains. The prognostic ability of the investigated factors may have been confirmed, but substantial heterogeneity between the studies was present and the effect sizes were in general fairly low, explaining only a limited part of the variance of outcome. Further research is naturally warranted to identify more important prognostic factors. Ultimately, this body of evidence can contribute to the development of clinical prediction models, which, in turn, will generate a basis for the future optimization of multidisciplinary biopsychosocial rehabilitation in chronic pain.

## Supplementary Material

SUPPLEMENTARY MATERIAL

Supplemental Digital Content is available for this article. Direct URL citations appear in the printed text and are provided in the HTML and PDF versions of this article on the journal's website, www.clinicalpain.com.
