# Supplementary material for: Prognostic Factors for Physical Functioning After Multidisciplinary Rehabilitation in Patients With Chronic Musculoskeletal Pain: A Systematic Review and Meta-Analysis
Source: Clin J Pain. 2018 Oct 26;35(2):148–73. doi: 10.1097/AJP.0000000000000669 (PMC6343958; doi:10.1097/AJP.0000000000000669)
Supplement: SUPPLEMENTARY MATERIAL [file ajp-35-148-s001.pdf]

Karolinska Institutet

1. Medline (Ovid)

1. Pain/
2. Chronic Pain/
3. exp Musculoskeletal Pain/
4. exp Back Pain/
5. Neck Pain/
6. Shoulder Pain/
7. Headache/
8. Nociceptive Pain/
9. Pain, Intractable/
10. Myalgia/
11. Myofascial Pain Syndromes/
12. Fibromyalgia/
13. Whiplash Injuries/
14. (chronic pain or long-lasting pain or intermittent pain or long-term pain or persistent pain or intractable pain or musculoskeletal pain or chronic muscular pain or nociceptive pain or shoulder pain or neck pain or whiplash or back pain or low back pain or generalized pain or widespread pain or fibromyalgia or myofascial pain syndrome or myalgia or idiopathic pain or diffuse pain or aspecific pain or non-specific pain or musculoskeletal pain syndrome or chronic pain syndrome or somatoform pain syndrome or non-cancer pain or non-malignant pain or benign pain).tw,kf.
15. or/1-14
16. Pain Management/
17. Pain clinics/
18. Rehabilitation/
19. Rehabilitation, Vocational/
20. Rehabilitation.fs.
21. Rehabilitation Centers/
22. Patient Care Team/
23. ((multidisciplinary or multiprofessional or multimodal or interprofessional or inter-professional or interdisciplinary or inter-disciplinary or team or biopsychosocial) adj3 (rehabilitation or treatment* or intervention* or regimen* or management or clinic* or therap* or program*)).tw,kf.
24. functional restoration program.tw,kf.
25. or/16-24
26. Forecasting/
27. Prognosis/
28. exp Probability/
29. exp Epidemiologic Factors/
30. exp Regression Analysis/
31. (predict* or forecasting or prognos* or probability or determinant* or moderator or effect modifi* or Regression Analys?s or Logistic Regression or Logistic Model* or Odds Ratio or Risk Ratio or relative risk or risk factor* or protective factor* or Cox model* or Hazard* model* or hazard ratio).tw,kf.
32. or/26-31
33. "Outcome and Process Assessment (Health Care)"/
34. Outcome Assessment Health Care/
35. Patient Outcome Assessment/
36. Treatment Outcome/
37. Program Evaluation/
38. Disability Evaluation/
39. Follow-up Studies/
40. (treatment outcome* or treatment effect* or treatment result* or treatment efficacy or intervention* outcome* or rehabilitation outcome* or health outcome* or patient-relevant outcome* or outcome assessment* or outcome measurement* or clinical effect* or clinical efficacy or clinical trial or randomized controlled trial or observational study or follow-up study or longitudinal study or prospective study or retrospective study or cohort study).tw,kf.
41. or/33-40
42. 15 and 25 and 32 and 41
43. exp animals/ not exp humans/
44. 42 not 43
45. remove duplicates from 44
46. limit 45 to "review articles"
47. 45 not 46
48. limit 47 to yr="1980 -Current"

Karolinska Institutet

2. Cinahl (Ebsco)

#	Query
S37	S14 AND S22 AND S29 AND S36
S36	S30 OR S31 OR S32 OR S33 OR S34 OR S35
S35	TI ("treatment outcome*" or "treatment effect*" or "treatment result*" or "treatment efficacy" or "intervention outcome*" or "rehabilitation outcome*" or "health outcome*" or "patient relevant outcome*" or "outcome assessment*" or "outcome measurement*" or "clinical effect*" or "clinical efficacy" or "clinical trial" or "randomized controlled trial" or "observational study" or "follow-up study" or "longitudinal study" or "prospective study" or "retrospective study" or "cohort study")OR AB ("treatment outcome*" or "treatment effect*" or "treatment result*" or "treatment efficacy" or "intervention outcome*" or "rehabilitation outcome*" or "health outcome*" or "patient relevant outcome*" or "outcome assessment*" or "outcome measurement*" or "clinical effect*" or "clinical efficacy" or "clinical trial" or "randomized controlled trial" or "observational study" or "follow-up study" or "longitudinal study" or "prospective study" or "retrospective study" or "cohort study")
S34	(MH "Disability Evaluation")
S33	(MH "Program Evaluation")
S32	(MH "Treatment Outcomes")
S31	(MH "Outcome Assessment")
S30	(MH "Outcomes (Health Care)")
S29	S23 OR S24 OR S25 OR S26 OR S27 OR S28
S28	TI (predict* or forecasting or prognos* or probability or determinant* or moderator or "effect modifi*" or "Regression Analys?s" or "Logistic Regression" or "Logistic Model" or "Odds Ratio" or "Risk Ratio" or "relative risk" or "risk factor" or "protective factor" or "Cox model" or "Hazard model" or "hazard ratio") OR AB (predict* or forecasting or prognos* or probability or determinant* or moderator or "effect modifi*" or "Regression Analys?s" or "Logistic Regression" or "Logistic Model" or "Odds Ratio" or "Risk Ratio" or "relative risk" or "risk factor" or "protective factor" or "Cox model" or "Hazard model" or "hazard ratio")
S27	(MH "Regression+")
S26	(MH "Probability")
S25	(MH "Prognosis")
S24	(MH "Predictive Research")
S23	(MH "Forecasting (Research)")
S22	S15 OR S16 OR S17 OR S18 OR S19 OR S20 OR S21
S21	TI ((multidisciplinary OR multiprofessional OR multimodal OR interprofessional OR inter-professional OR interdisciplinary OR inter-disciplinary OR team OR biopsychosocial) N2 (rehabilitation OR treatment* OR intervention* OR regimen* OR management OR clinic* OR therap* OR program*)) OR AB ((multidisciplinary OR multiprofessional OR multimodal OR interprofessional OR inter-professional OR interdisciplinary OR inter-disciplinary OR team OR biopsychosocial) N2 (rehabilitation OR treatment* OR intervention* OR regimen* OR management OR clinic* OR therap* OR program*))
S20	(MH "Multidisciplinary Care Team")
S19	(MH "Rehabilitation Centers")
S18	(MH "Rehabilitation, Psychosocial")

S17	(MH "Rehabilitation, Vocational")
S16	(MH "Rehabilitation")
S15	(MH "Pain Clinics")
S14	S1 OR S2 OR S3 OR S4 OR S5 OR S6 OR S7 OR S8 OR S9 OR S10 OR S11 OR S12 OR S13
S13	TI ((”chronic pain” or ”long-lasting pain” or ”intermittent pain” or ”long-term pain” or ”persistent pain” or ”intractable pain” or ”musculoskeletal pain” or ”chronic muscular pain” or ”nociceptive pain” or ”shoulder pain” or ”neck pain” or ”whiplash” or ”back pain” or ”low back pain” or ”generalized pain” or ”widespread pain” or ”fibromyalgia” or ”myofascial pain syndrome” or ”myalgia” or ”idiopathic pain” or ”diffuse pain” or ”aspecific pain” or ”non-specific pain” or ”musculoskeletal pain syndrome” or ”chronic pain syndrome” or ”somatoform pain syndrome” or ”non-cancer pain” or ”non-malignant pain” or ”benign pain”)) OR AB ((”chronic pain” or ”long-lasting pain” or ”intermittent pain” or ”long-term pain” or ”persistent pain” or ”intractable pain” or ”musculoskeletal pain” or ”chronic muscular pain” or ”nociceptive pain” or ”shoulder pain” or ”neck pain” or ”whiplash” or ”back pain” or ”low back pain” or ”generalized pain” or ”widespread pain” or ”fibromyalgia” or ”myofascial pain syndrome” or ”myalgia” or ”idiopathic pain” or ”diffuse pain” or ”aspecific pain” or ”non-specific pain” or ”musculoskeletal pain syndrome” or ”chronic pain syndrome” or ”somatoform pain syndrome” or ”non-cancer pain” or ”non-malignant pain” or ”benign pain”))
S12	(MH "Whiplash Injuries")
S11	(MH "Fibromyalgia")
S10	(MH "Myofascial Pain Syndromes")
S9	(MH "Muscle Pain")
S8	(MH "Nociceptive Pain")
S7	(MH "Headache")
S6	(MH "Shoulder Pain")
S5	(MH "Neck Pain")
S4	(MH "Low Back Pain")
S3	(MH "Back Pain")
S2	(MH "Chronic Pain")
S1	(MH "Pain")

3. PsychINFO (Ovid)

1. Pain/
2. Chronic Pain/
3. exp Musculoskeletal Pain/
4. exp Back Pain/
5. Neck Pain/
6. Shoulder Pain/
7. Headache/
8. Nociceptive Pain/
9. Pain, Intractable/
10. Myalgia/
11. Myofascial Pain Syndromes/
12. Fibromyalgia/
13. Whiplash Injuries/
14. (chronic pain or long-lasting pain or intermittent pain or long-term pain or persistent pain or intractable pain or musculoskeletal pain or chronic muscular pain or nociceptive pain or shoulder pain or neck pain or whiplash or back pain or low back pain or generalized pain or widespread pain or fibromyalgia or myofascial pain syndrome or myalgia or idiopathic pain or diffuse pain or aspecific pain or non-specific pain or musculoskeletal pain syndrome or chronic pain syndrome or somatoform pain syndrome or non-cancer pain or non-malignant pain or benign pain).tw,kf.
15. or/1-14
16. Pain Management/
17. Pain clinics/
18. Rehabilitation/
19. Rehabilitation, Vocational/
20. Rehabilitation.fs.
21. Rehabilitation Centers/
22. Patient Care Team/
23. ((multidisciplinary or multiprofessional or multimodal or interprofessional or inter-professional or interdisciplinary or inter-disciplinary or team or biopsychosocial) adj3 (rehabilitation or treatment* or intervention* or regimen* or management or clinic* or therap* or program*)).tw,kf.
24. functional restoration program.tw,kf.
25. or/16-24
26. Forecasting/
27. Prognosis/
28. exp Probability/
29. exp Epidemiologic Factors/
30. exp Regression Analysis/
31. (predict* or forecasting or prognos* or probability or determinant* or moderator or effect modifi* or Regression Analys?s or Logistic Regression or Logistic Model* or Odds Ratio or Risk Ratio or relative risk or risk factor* or protective factor* or Cox model* or Hazard* model* or hazard ratio).tw,kf.
32. or/26-31
33. "Outcome and Process Assessment (Health Care)"/
34. Outcome Assessment Health Care/
35. Patient Outcome Assessment/
36. Treatment Outcome/
37. Program Evaluation/
38. Disability Evaluation/
39. Follow-up Studies/
40. (treatment outcome* or treatment effect* or treatment result* or treatment efficacy or intervention* outcome* or rehabilitation outcome* or health outcome* or patient-relevant outcome* or outcome assessment* or outcome measurement* or clinical effect* or clinical efficacy or clinical trial or randomized controlled trial or observational study or follow-up study or longitudinal study or prospective study or retrospective study or cohort study).tw,kf.
41. or/33-40
42. 15 and 25 and 32 and 41
43. exp animals/ not exp humans/
44. 42 not 43
45. remove duplicates from 44
46. limit 45 to "review articles"
47. 45 not 46
48. limit 47 to yr="1980 -Current"

4. Embase (embase.com)

#40	**#38** NOT **#39** AND [1980-2015]/py
#39	**#37** NOT ([animals]/lim NOT [humans]/lim) AND [systematic review]/lim
#38	**#37** NOT ([animals]/lim NOT [humans]/lim)
#37	**#16** AND **#24** AND **#30** AND **#36**
#36	**#31** OR **#32** OR **#33** OR **#34** OR **#35**
#35	(**treatment** NEXT/1 **outcome**):ab,ti OR (**treatment** NEXT/1 **effect**):ab,ti OR (**treatment** NEXT/1 **result**):ab,ti OR (**treatment** NEXT/1 **efficacy**):ab,ti OR (**intervention** NEXT/1 **outcome**):ab,ti OR (**rehabilitation** NEXT/1 **outcome**):ab,ti OR (**health** NEXT/1 **outcome**):ab,ti OR (**'patient relevant'** NEXT/1 **outcome**):ab,ti OR (**outcome** NEXT/1 **assessment**):ab,ti OR (**outcome** NEXT/1 **measurement**):ab,ti OR (**clinical** NEXT/1 **effect**):ab,ti OR (**clinical** NEXT/1 **efficacy**):ab,ti OR (**clinical** NEXT/1 **trial**):ab,ti OR (**randomized** NEXT/1 **controlled** NEXT/1 **trial**):ab,ti OR (**observational** NEXT/1 **study**):ab,ti OR (**'follow up'** NEXT/1 **study**):ab,ti OR (**longitudinal** NEXT/1 **study**):ab,ti OR (**prospective** NEXT/1 **study**):ab,ti OR (**retrospective** NEXT/1 **study**):ab,ti OR (**cohort** NEXT/1 **study**):ab,ti
#34	**'evaluation and follow up'**/exp
#33	**'disability'**/exp
#32	**'program evaluation'**/de
#31	**'treatment outcome'**/exp
#30	**#25** OR **#26** OR **#27** OR **#28** OR **#29**
#29	**predict***:ab,ti OR **forecasting**:ab,ti OR **prognos***:ab,ti OR **probability**:ab,ti OR **determinant***:ab,ti OR **moderator**:ab,ti OR (**effect** NEXT/1 **modifi***):ab,ti OR (**regression** NEXT/1 **analysis**):ab,ti OR (**logistic** NEXT/1 **regression**):ab,ti OR (**logistic** NEXT/1 **model***):ab,ti OR (**odds** NEXT/1 **ratio**):ab,ti OR (**risk** NEXT/1 **ratio**):ab,ti OR (**relative** NEXT/1 **risk**):ab,ti OR (**risk** NEXT/1 **factor***):ab,ti OR (**protective** NEXT/1 **factor***):ab,ti OR (**cox** NEXT/1 **model**):ab,ti OR (**hazard** NEXT/1 **model**):ab,ti OR (**hazard** NEXT/1 **ratio**):ab,ti
#28	**'regression analysis'**/exp
#27	**'epidemiology'**/de
#26	**'prognosis'**/de
#25	**'prediction'**/de
#24	**#17** OR **#18** OR **#19** OR **#20** OR **#21** OR **#22** OR **#23**
#23	((**multidisciplinary** OR **multiprofessional** OR **multimodal** OR **interprofessional** OR **'inter professional'** OR **interdisciplinary** OR **'inter disciplinary'** OR **team** OR **biopsychosocial**) NEAR/3 (**rehabilitation** OR **treatment*** OR **intervention*** OR **regimen*** OR **management** OR **clinic*** OR **therap*** OR **program***)):ab,ti
#22	**'rehabilitation center'**/de
#21	**'vocational rehabilitation'**/de
#20	**'psychosocial rehabilitation'**/de
#19	**'functional training'**/de
#18	**'rehabilitation'**/de
#17	**'pain clinic'**/de
#16	**#1** OR **#2** OR **#3** OR **#4** OR **#5** OR **#6** OR **#7** OR **#8** OR **#9** OR **#10** OR **#11** OR **#12** OR **#13** OR **#14** OR **#15**

#15	'chronic pain':ab,ti OR 'long-lasting pain':ab,ti OR 'intermittent pain':ab,ti OR 'long-term pain':ab,ti OR 'persistent pain':ab,ti OR 'intractable pain':ab,ti OR 'musculoskeletal pain':ab,ti OR 'chronic muscular pain':ab,ti OR 'nociceptive pain':ab,ti OR 'shoulder pain':ab,ti OR 'neck pain':ab,ti OR 'whiplash':ab,ti OR 'back pain':ab,ti OR 'low back pain':ab,ti OR 'generalized pain':ab,ti OR 'widespread pain':ab,ti OR 'fibromyalgia':ab,ti OR 'myofascial pain syndrome':ab,ti OR 'myalgia':ab,ti OR 'idiopathic pain':ab,ti OR 'diffuse pain':ab,ti OR 'aspecific pain':ab,ti OR 'non-specific pain':ab,ti OR 'musculoskeletal pain syndrome':ab,ti OR 'chronic pain syndrome':ab,ti OR 'somatoform pain syndrome':ab,ti OR 'non-cancer pain':ab,ti OR 'non-malignant pain':ab,ti OR 'benign pain':ab,ti
#14	'whiplash injury'/de
#13	'fibromyalgia'/de
#12	'myofascial pain'/de
#11	'myalgia'/de
#10	'intractable pain'/de
#9	'nociceptive pain'/de
#8	'headache'/de
#7	'shoulder pain'/de
#6	'neck pain'/de
#5	'low back pain'/de
#4	'backache'/de
#3	'musculoskeletal pain'/de
#2	'chronic pain'/de
#1	'pain'/de

5. Web of Science Core Collection

#5	#4 AND #3 AND #2 AND #1<br>*DocType=All document types; Language=All languages;*
#4	TS=("treatment outcome" or ”treatment effect” or ”treatment result” or ”treatment efficacy” or ”intervention outcome” or ”rehabilitation outcome” or ”Health outcome” or ”patient-relevant outcome” or ”outcome assessment” or ”outcome measurement” or ”clinical effect” or ”clinical efficacy” or ”clinical trial” or ”randomized controlled trial” or ”observational study” or ”follow-up study” or ”longitudinal study” or ”prospective study” or ”retrospective study” or ”cohort study”)<br>*DocType=All document types; Language=All languages;*
#3	TS=(predict* or forecasting or prognos* or probability or determinant* or moderator or ”effect modifi” or ”Regression Analys?s” or ”Logistic Regression” or ”Logistic Model” or ”Odds Ratio” or ”Risk Ratio” or ”relative risk” or ”risk factor” or ”protective factor” or ”Cox model” or ”Hazard model” or ”hazard ratio”)<br>*DocType=All document types; Language=All languages;*
#2	TS=((multidisciplinary or multiprofessional or multimodal or interprofessional or inter-professional or interdisciplinary or inter-disciplinary or team or biopsychosocial) NEAR/3 (rehabilitation or treatment* or intervention* or regimen* or management or clinic* or therap* or program*))<br>*DocType=All document types; Language=All languages;*
#1	TS=(”chronic pain” or ”long-lasting pain” or ”intermittent pain” or ”long-term pain” or ”persistent pain” or ”intractable pain” or ”musculoskeletal pain” or ”chronic muscular pain” or ”nociceptive pain” or ”shoulder pain” or ”neck pain” or ”whiplash” or ”back pain” or ”low back pain” or ”generalized pain” or ”widespread pain” or ”fibromyalgia” or ”myofascial pain syndrome” or ”myalgia” or ”idiopathic pain” or ”diffuse pain” or ”aspecific pain” or ”non-specific pain” or ”musculoskeletal pain syndrome” or ”chronic pain syndrome” or ”somatoform pain syndrome” or ”non-cancer pain” or ”non-malignant pain” or ”benign pain”)<br>*DocType=All document types; Language=All languages;*

Karolinska Institutet

6. Cochrane Library (Wiley)/ Central

#1	("chronic pain" or "long-lasting pain" or "intermittent pain" or "long-term pain" or "persistent pain" or "intractable pain" or "musculoskeletal pain" or "chronic muscular pain" or "nociceptive pain" or "shoulder pain" or "neck pain" or "whiplash" or "back pain" or "low back pain" or "generalized pain" or "widespread pain" or "fibromyalgia" or "myofascial pain syndrome" or "myalgia" or "idiopathic pain" or "diffuse pain" or "aspecific pain" or "non-specific pain" or "musculoskeletal pain syndrome" or "chronic pain syndrome" or "somatoform pain syndrome" or "non-cancer pain" or "non-malignant pain" or "benign pain"):ab,ti
2	((multidisciplinary or multiprofessional or multimodal or interprofessional or inter-professional or interdisciplinary or inter-disciplinary or team or biopsychosocial) near/3 (rehabilitation or treatment* or intervention* or regimen* or management or clinic* or therap* or program*)):ab,ti
#3	("Pain management" or "pain clinic" or "rehabilitation center"):ab,ti
#4	#2 or #3
#5	(predict* or forecasting or prognos* or probability or determinant* or moderator or "effect modifi*" or "Regression Analys?s" or "Logistic Regression" or "Logistic Model" or "Odds Ratio" or "Risk Ratio" or "relative risk" or "risk factor" or "protective factor" or "Cox model" or "Hazard model" or "hazard ratio"):ab,ti
#6	("treatment outcome*" or "treatment effect*" or "treatment result*" or "treatment efficacy" or "intervention outcome*" or "rehabilitation outcome*" or "health outcome*" or "patient relevant outcome*" or "outcome assessment*" or "outcome measurement*" or "clinical effect*" or "clinical efficacy" or "clinical trial" or "randomized controlled trial" or "observational study" or "follow-up study" or "longitudinal study" or "prospective study" or "retrospective study" or "cohort study"):ab,ti
#7	#1 and #4 and #5 and #6
